# Smoking, tooth loss and oral hygiene practices have significant and site-specific impacts on the microbiome of oral mucosal surfaces: a cross-sectional study

**DOI:** 10.1080/20002297.2023.2263971

**Published:** 2023-10-02

**Authors:** Sheila Galvin, Sviatlana Anishchuk, Claire M. Healy, Gary P. Moran

**Affiliations:** a Division of Oral and Maxillofacial Surgery, Oral Medicine and Oral Pathology, School of Dental Science, Trinity College Dublin, Dublin Dental University Hospital, Dublin, Ireland; b Division of Oral Biosciences, School of Dental Science, Trinity College Dublin, Dublin Dental University Hospital, Dublin, Ireland

**Keywords:** Oral mucosa, microbiome, 16S rRNA, smoking, tooth loss, oral hygiene

## Abstract

We investigated bacterial colonisation patterns of healthy mucosa (buccal, tongue, palate and floor of mouth) in a cohort of adults in order to determine how smoking, tooth loss, plaque levels and oral hygiene practices impacted on mucosal colonisation. A total of 322 swabs were recovered from 256 participants, of whom 46% were current smokers. We analysed colonization by sequencing the V1-V3 regions of the 16S rRNA gene. Palate and tongue microbiomes generally exhibited greater biodiversity than buccal and floor of mouth. Although *Neisseria*, *Lautropia* and *Haemophilus* spp. showed reduced abundance in smokers, buccal mucosa specifically showed a significant increase in *Prevotella* spp., whereas tongue and floor of mouth tended towards increased abundance of *Streptococcus* spp. Unexpectedly, tooth brushing frequency had a greater impact on mucosal community structure than plaque levels. Tooth loss was associated with significant reductions in mucosal biodiversity and had site-specific impacts, with buccal communities showing increased abundance of periodontitis-associated species and *Rothia mucilaginosa*, whereas tongue communities exhibited increased abundance of several streptococcal OTUs and reduced abundance of *Haemophilus* spp. This study highlights the complex relationship between mucosal colonisation and host factors, highlighting the need for careful consideration of these factors in mucosal microbiome studies.

## Introduction

The oral microbiome has undergone intense investigation to understand its core composition and role in common human diseases, such as dental decay (caries) and gum disease (periodontitis) [1–4]. Early culture-independent, molecular techniques using 16S rRNA cloning demonstrated that this microbiome was highly diverse and site specific, with a high proportion of uncultured species [5–7]. With the advent of next-generation sequencing, the Human Microbiome Consortium sampled the oral cavity of North American adults and characterised a rich bacterial community that differed between intraoral niches, with a conserved core microbiome that was shared between individuals [8–10]. Amplification and sequencing of the variable regions of the 16S rRNA gene is still the method of choice in studies, and the development of the aerodigestive habitat-specific expanded Human Oral Microbiome Database (eHOMD) has allowed species level or supra-species level classification of 16S rRNA reads [11,12]. Further, fine-scale subspecies typing can be achieved by oligotyping, which was used by Eren et al. to show that different oligotypes of *Streptococcus*, *Neisseria* and *Veillonella* (which could not be resolved using traditional analysis) exhibited very different distributions among oral sites [13].

Although our understanding of oral ecology has greatly improved, the factors that result in interindividual variability are still poorly understood. We know that events early in life such as infant feeding methods and antibiotic usage can have long-term impacts on oral microbiome structure and that events such as tooth eruption play an important role in maturation of the oral microbiome [2,14–16]. Variation in the adult microbiome may be influenced by smoking, oral hygiene, diet (notably sugar consumption) and periodontal health [3,16–21]. Many of these studies analyse the salivary microbiome as a representation of the oral microbiome [22], or in the case of studies examining oral hygiene and periodontal health, focus on plaque composition [17,18,23]. The influence of host and environmental factors on the microbiome of mucosal surfaces is less well understood and given the site specificity of the oral microbiome, the site-specific impacts of these factors are also poorly understood. As an example, the largest study to date of smoking and the oral microbiome focuses solely on saliva [17]. The impact of smoking on mucosal surfaces has been reported, but the studies are relatively small and often have low proportions of current smokers [24,25].

The current study was designed to examine the microbiome of four distinct oral niches, namely buccal, tongue, palate and floor of mouth (FOM). Healthy mucosal sites were sampled from patients attending for routine dental care and from patients attending an oral dysplasia clinic, with the latter group containing a high proportion of smokers and alcohol users with relatively high levels of tooth-loss. Multivariate analyses were used to investigate the impacts of variables including smoking, tooth loss and oral hygiene practices on the oral microbiome and to determine if any site-specific impacts could be identified.

## Materials and methods

### Sample collection

Ethical Approval for this study was granted by the Joint Hospitals’ Research Ethics Committee, Tallaght Hospital, Dublin (Ref: 2017–11-Chairman’s actions [7]). Following written informed patient consent, mucosal swabs were collected at a single site, the Dublin Dental University Hospital. Recruitment was carried out among patients attending general dental clinics for routine check-ups (*n* = 61) and from patients attending the oral medicine clinic (*n* = 195). Only healthy mucosal tissue was sampled. Swabs were taken using sterile polyurethane foam swabs (CultureSwab EZ, BD) by a dental hygienist by gently rubbing the surface of the mucosa 10 times with rotation of the swab. Samples were recovered at least 1 h after eating, oral hygiene or mouthwash use. Participants were recorded as current smokers, former smokers or never smokers and alcohol consumption was recorded as units of alcohol consumed per week. Oral health of the participants was recorded including number of teeth, plaque levels (determined using the simplified oral hygiene index, OHI-S [26]), tooth brushing frequency, mouthwash use and denture wearing.

Patients having taken antibiotics or used topical steroids intra-orally in the past 3 months were excluded along with patients with diabetes mellitus, Crohn’s disease, ulcerative colitis, current viral infection (cold/flu), or history of gastrointestinal malignancy. A total of 322 oral mucosal swabs were collected as detailed in Table 1. For this analysis, a minimum sample size of 235 was determined based on previous data [27]. Using the chi-square test to determine if there is an association between mucosal sites (four categories) and a variable with three levels (e.g. never, current and former smoker) an effect size of 0.25 can be identified assuming an estimated power of 0.8, level of significance 0.05.

**Table 1.** Mucosal sites sampled (percentages in brackets).

Anatomical areas	Sub-sites	Number (%)
Floor of mouth (Fom)	Floor of mouth, ventral tongue	43 (13.3%)
Buccal (Buc)	Buccal mucosa, Gingiva	128 (39.8%)
Tongue (Ton)	Dorsum, lateral border tongue	115 (35.7%)
Palate (Pal)	Palate	36 (11.2%)
Total		322 (100%)

### DNA extraction and sequencing

Swabs were immediately stored at −80°C for a maximum of 6 weeks prior to DNA extraction. DNA was extracted from mucosal swabs using the MasterPure Complete DNA/RNA Purification Kit (Epicentre Biotechnologies). The manufacturer’s extraction protocol was supplemented with an additional incubation step with Ready-Lyse lysozyme (Epicentre Biotechnologies) and a bead disruption step, as described by Amer et al. [27]. The DNA was resuspended in a Tris-EDTA buffer (pH 7.5) and stored at −80°C. Amplification of the V1–V3 region of the 16S rRNA gene was carried out using the primers 27F-YM and 519 R (27F-YM: 5′-AGAGTTTGATYMTGGCTCAG; 519 R: 5′-GWATTACCGCGGCKGCTG) [28] and was performed in duplicate using separate template dilutions (1:1 and 1:10). Amplification and sequencing was performed by Integrated Microbiome Resources (IMR; Dalhousie, Canada) according to their standardised, in-house protocols described by Comeau et al. [29] and detailed here: https://www.protocols.io/workspaces/integrated-microbiome-resource-imr/publications. Paired end sequencing was performed using the Illumina 600 cycle MiSeq reagent kit. All sequence data has been submitted to the NCBI sequence read archive (SRA), submission SUB13676038, BioProject PRJNA994083.

### Data analysis

Bacterial 16S rRNA sequences were processed and filtered using Dada2 with the following parameters: maxN = 0, maxEE = c(2,5) and truncQ = 2 [30]. The 10 terminal bases were trimmed from reverse reads due to the drop in sequence quality. Following error estimation and correction using the Dada2 algorithm, paired reads were merged and chimeras were removed following their identification with the removeBimeraDenovo command. Taxonomy was assigned using the Human Oral Microbiome Database (HOMD) classifications (eHOMD 16S rRNA RefSeq version 15.1) [7]. The packages Phyloseq [31] and MicrobiotaProcess [32] were used to calculate the alpha diversity indices for each sample and for comparison of beta diversity (community structure) [31]. Further visualisations (biplots) and analysis of community structure, including PERMANOVA, were conducted using the package MicroViz in R studio [33,34]. Multivariate analysis was conducted with MaAsLin 2 using relative abundance data and the default linear models (LM) setting with log transformation of the data [34]. MaAsLin 2 was used to control for the influence of patient variables such as smoking, oral hygiene and tooth loss and to exclude any possible associations between them. We also included the clinic attended (either oral medicine clinic or general dental clinic) as a random effect to test if this had a significant impact on the results. The indicated patient metadata categories were used as fixed-effects with ‘healthy’ variables set as the reference (e.g. never smokers, 0 alcohol units/week, good oral hygiene, <5 missing teeth, brushing >1 per day) [34]. MaAsLin2 generates tabular outputs (see supplementary tables) detailing a coefficient of variance from the reference and adjusted *p* values (q-values; Benjamini-Hochberg FDR). Heatmaps show the 50 most significant values (default Padj < 0.25) with the effect size calculated as (-log (q-value) * (coefficient)).

## Results

Swabs from healthy oral mucosal surfaces were analysed to investigate the influence of mucosal site, age, gender, smoking, alcohol consumption, plaque levels, tooth loss and denture wear on the microbiome. A total of 322 sites were sampled from 256 patients. Of these patients, 195 were attending the oral medicine clinic and 61 were attending general dental clinics at the Dublin Dental Hospital. Oral sites swabbed included four main anatomical areas: floor of mouth (FOM), buccal, tongue and palate (Table 1). Buccal and tongue samples were recovered from almost all participants, where healthy mucosa was present. A proportion of patients were also sampled at the palate (*n* = 36) and floor of mouth (FOM, *n* = 43). There was no statistical difference in the proportions of sites swabbed in the oral medicine and general dental patients (Table S1). However, those attending the oral medicine clinic were more likely to smoke, drink alcohol, had poorer oral hygiene and had fewer teeth compared to those attending the general dental clinics (Table S1).

**Figure 1.**
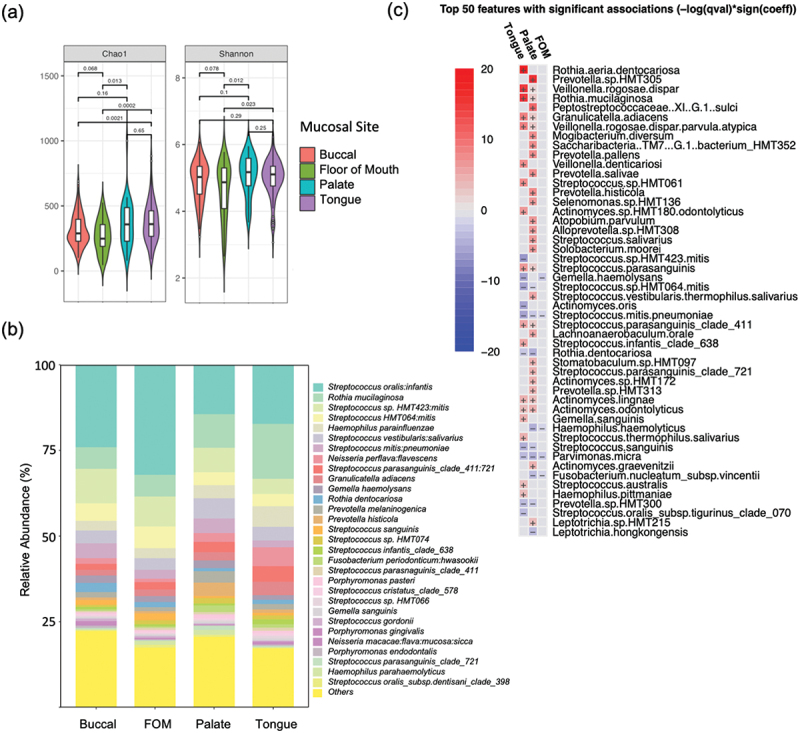
Analysis of healthy mucosal microbiomes by site. (a) Alpha diversity values for buccal, floor of mouth (FOM), palate and tongue communities. (b) Bar plot showing relative abundance (%) of the 30 most abundant species. (c) Results of multivariate analysis carried out using MaAsLin2 comparing FOM, tongue and palate communities with buccal mucosa.

Analysis of alpha diversity showed that tongue and palate communities exhibited similar Chao1 richness values and Shannon diversity index values (Figure 1a). Communities from the FOM and buccal regions had significantly reduced Chao1 values compared to tongue communities (Figure 1a). Biodiversity was lowest in FOM communities, which was significantly reduced compared to both palate (Shannon and Simpson) and tongue (Simpson) (Figure 1a and Fig. S1a).

Although different mucosal sites had similar core microbiomes, mucosal site was found to have a significant influence on microbial community structure. Buccal and FOM sites had the greatest proportions of *Streptococcus* species, tongue sites showed increased abundance of *Rothia* and *Neisseria* species, while palatal sites showed increased abundances of *Prevotella* species (Figure 1b). PCA (principal component analysis) of community membership by site also highlighted these genera as being responsible for differences in community structure (Fig. S1b). Inter-site comparisons of Bray-Curtis dissimilarity values using pairwise PERMANOVA confirmed this, showing significant differences in community structure between all sites with the exception of buccal and floor of mouth communities (Table 2).

**Table 2.** Pairwise PERMANOVA results for mucosal site comparisons.

Site comparisons	p value	Adjusted *p* value
Tongue vs Palate	0.004	0.004
Tongue vs Buccal	0.0001	0.0002
Tongue vs FOM	0.0001	0.0002
Palate vs Buccal	0.0001	0.0002
Palate vs FOM	0.0001	0.0002
Buccal vs FOM	0.13	0.13

MaAsLin2 analysis was used to carry out a multivariate analysis to investigate which species showed significant differences in abundance at each site. Using buccal mucosa as reference, 59 species showed significant changes (Padj < 0.05) in abundance, with 28 on the tongue, 30 on the palate, but only one on FOM (Figure 1c and Table S2). The most significant changes at tongue sites were increased abundances of *Rothia* spp. including *R. mucilaginosa* and *R. aeria:dentocariosa*, *Veillonella* species and *Actinomyces* species. Abundances of *Granulicatella adiacens* and *S. parasanguinis* were also significantly increased (Figure 1c and Table S1). Eleven species showed reduced abundance at tongue sites including OTUs related to *S. mitis* (Figure 1c and Table S1).

Significant changes at palate sites included increased abundance of six *Prevotella* species, four *Actinomyces* species and three *Streptococcus* species. Relative to both tongue and palate, buccal mucosa had significantly higher levels of *S. mitis, R. dentocariosa, S. sanguinis* and *P. micra* (Figure 1c and Table S2). Multivariate analysis showed that these major site-specific changes were independent of smoking, oral hygiene and tooth loss and whether the patient attended oral medicine or general dental clinics (Table S3).

## Smoking and alcohol consumption and the mucosal microbiome

**Figure 2.**
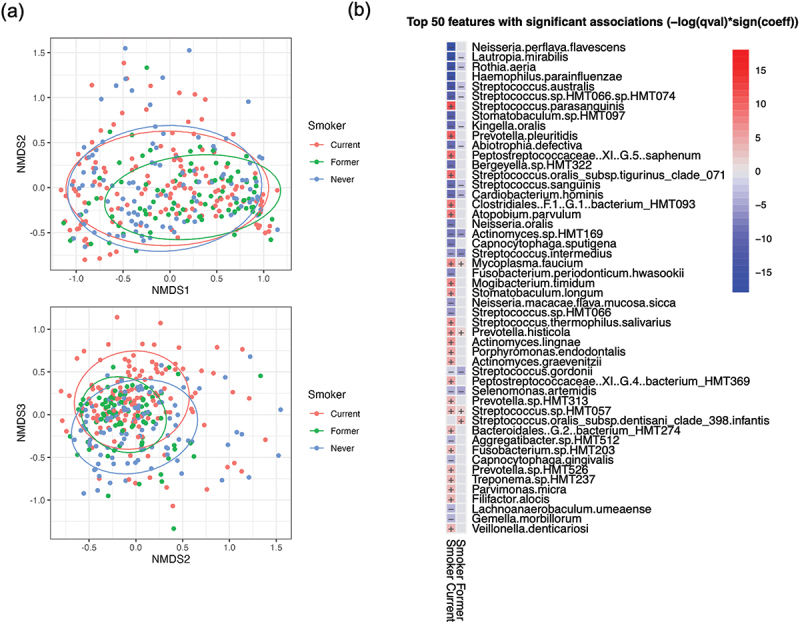
Analysis of the impact of smoking on the mucosal microbiome. (a) NMDS plots comparing community structure of samples based on Bray-Curtis dissimilarity index values. (b) Results of multivariate analysis carried out using MaAsLin2 with comparing the mucosal microbiomes of current and former smokers with never smokers.

Smoking was found to have a significant influence on the microbial community of mucosal surfaces. Although no significant changes in alpha diversity metrics were observed (Fig. S2), visualisation of community structure using NMDS plots of Bray-Curtis dissimilarity values showed separation of communities from current and never smokers, particularly along axis 3 (Figure 2a). Pairwise PERMANOVA comparisons showed that there were significant differences between the microbial communities of all three categories of smokers (Padj < 0.003; Table S4). MaAslin2 was used for multivariate analysis to identify which species were responsible for these differences. Twenty-eight species showed significantly decreased, and 29 species significantly increased abundances (all Padj < 0.05) associated with smoking (Figure 2b, Table S5). Smoking was associated with loss of species typically associated with healthy mucosa, including several species of *Neisseria* and *Streptococcus, L. mirabilis*, *R. aeria* and *H. parainfluenza*. Several Gram-negative species from the genera *Aggregatibacter*, *Kingella, Bergeyella, Fusobacterium and Capnocytophaga* also showed significant decreases in abundance in association with smoking (Figure 2b, Table S5). The species showing increased abundance associated with smoking were predominantly periodontitis-associated species from the genera *Prevotella, Porphyromonas, Fusobacterium, Tannerella, Parvimonas, Filifactor* and *Peptostreptococcaceae* (all Padj < 0.05; Figure 2b, Table S5).

**Figure 3.**
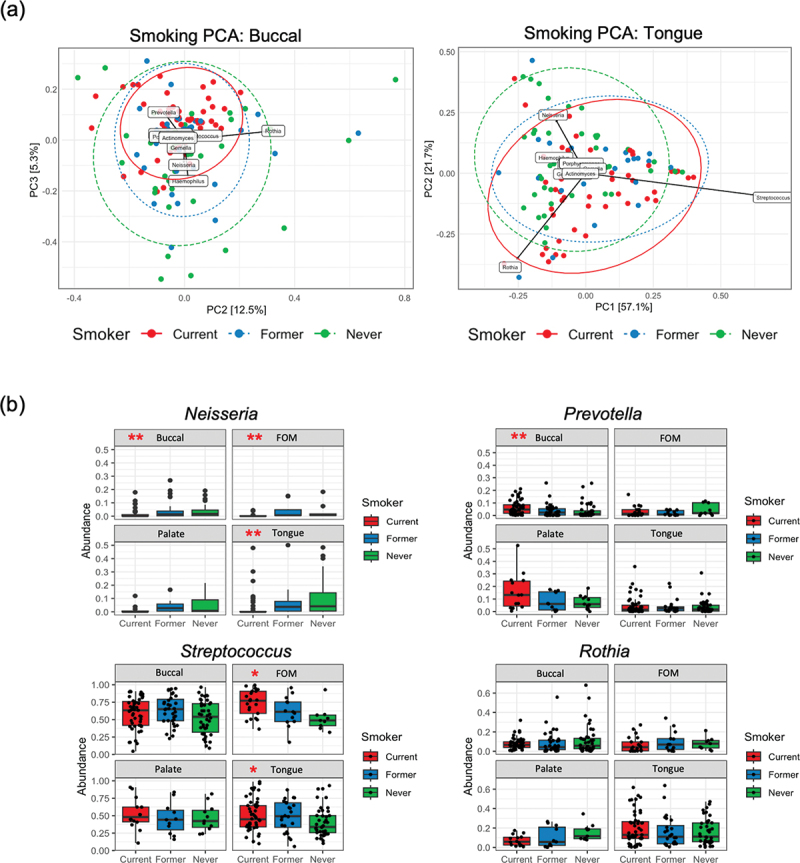
Site-specific effects of smoking on the mucosal microbiome. (a) PCA biplots comparing the genus level composition of buccal and tongue communities in smokers, former smokers and never smokers. Labelled arrows indicate the influence of specific genera in separating samples along the respective axis. (b) Box-plots showing relative abundance of *Neisseria, Prevotella, Streptococcus* and *Rothia* species in current, former and never smokers at each mucosal site. Significance values (MaAslin 2) are indicated by **(Padj <0.05) and *(Padj <0.2).

Examination of the impact of smoking at different mucosal sites revealed site-specific effects (Fig. S2b). *Neisseria, Lautropia* and *Kingella* spp. were all found to be reduced on tongue, buccal and floor of mouth in smokers. No significant changes were identified on the palate. Uniquely, buccal communities exhibited significant increased abundance of several genera associated with periodontal disease, including *Tannerella*, *Filifactor* and *Porphyromonas* (Fig. S2b). Analysis of buccal community composition showed that smoking was associated with a shift from *Neisseria* and *Haemophilus* towards communities rich in *Prevotella* (MaAsLin 2 Padj = 0.013; Figure 3a,b). PCA plots examining tongue communities also showed that smoking was associated with a shift from *Neisseria* rich communities towards communities with increased abundance of *Rothia* or *Streptococcus* (Figure 3a,b), although the increases in *Rothia* and *Streptococcus* were not highly significant (Figure 2d).

Alcohol consumption was not associated with any significant shifts in alpha or beta-diversity (*p* = 0.38) (Fig. S3). A multivariate analysis of smoking and alcohol consumption, while confirming the previous associations with smoking, identified that consumption of >20 units of alcohol per week resulted in increased levels of *C. concisus*, *P. melaninogenica* and reduced levels of *F. alocis* (Fig. S3).

## Oral hygiene and the mucosal microbiome

**Figure 4.**
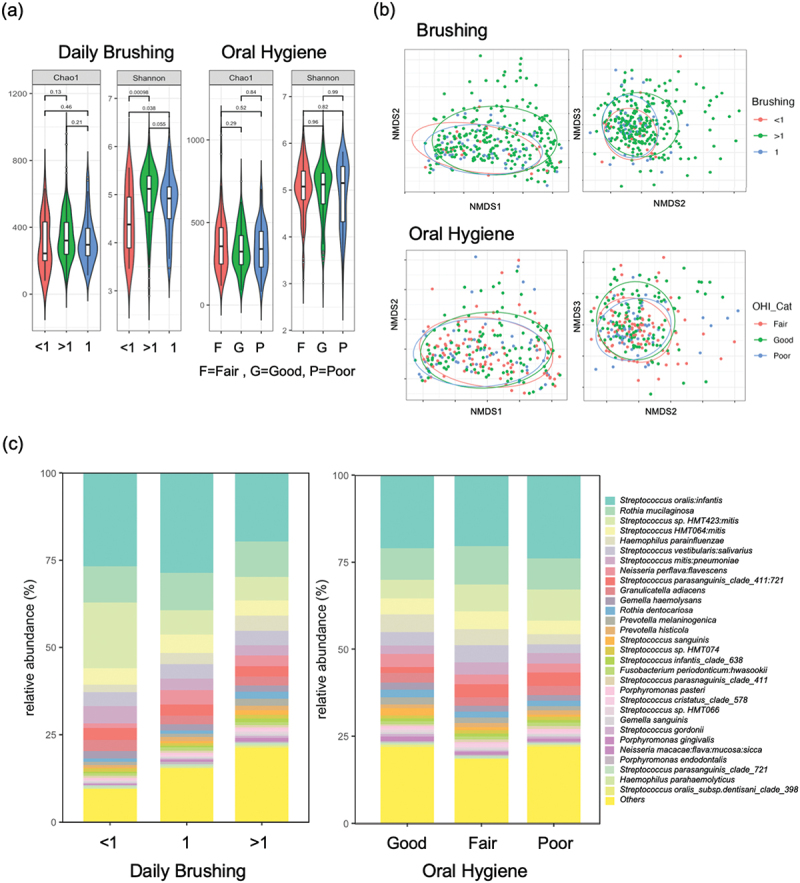
Impact of tooth brushing frequency (<1, 1 or > 1 per day) and oral hygiene (OHI-S index), on the mucosal microbiome. (a) Alpha diversity values according to daily brushing frequency and oral hygiene. (b) Community structure analysed by NMDS of Bray-Curtis dissimilarity values according to daily brushing frequency and oral hygiene. (c) Bar plot showing relative abundance (%) of the 30 most abundant species according to daily brushing frequency and oral hygiene.

We categorised our participants based on oral hygiene practices including tooth-brushing frequency (brushing <1, 1 or >1 per day), mouthwash use and on the basis of observed levels of plaque accumulation using the simplified oral hygiene index (OHI-S). The number of individuals who brushed frequently (>1 per day, *n* = 186) outnumbered those that brushed infrequently (≤1 Per day, *n* = 67). Brushing less than once per day was associated with reduced biodiversity as indicated by significantly reduced Shannon index values (Figure 4a). Analysis of community structure using NMDS plots comparing Bray-Curtis dissimilarity values demonstrated separation of samples where brushing occurred more than once per day (PERMANOVA *p* = 0.032; Figure 4b). Bar plots of relative species abundance indicated that infrequent brushing (≤1 Per day) was associated with higher levels of streptococci (Figure 4c). A multivariate analysis of brushing frequency with smoking showed that those who brushed infrequently (≤1 Per day) had significantly increased levels of the highly abundant OTU *S. oralis:infantis*, as well as *H. parahaemolyticus* and *G. elegans* (Table S6 and Fig. S4).

Measurement of plaque levels in dentate participants was assessed using the OHI-S index, which categorised the participants as having good (*n* = 77), fair (*n* = 77) and poor (*n* = 47) oral hygiene. Oral hygiene category had less of an impact on mucosal alpha diversity and population structure compared to brushing frequency (Figure 4a,b). It appeared that the shifts in population structure related to oral hygiene were not highly significant (Figure 4b; PERMANOVA Padj > 0.05). However, in a multivariate analysis (Table S6, Fig. S4), poor oral hygiene was associated with a significantly increased abundance of several plaque and periodontitis-associated species including *P. denticola* (Padj 0.09), *Leptotrichia* sp. HMT498 (Padj = 0.09), *Saccharibacteria* sp. HMT346 (Padj = 0.087), *Lachnospiraceae* sp. HMT100 (Padj = 0.023), *Tannerella* sp. HMT286 (Padj = 0.058) and *F. nucleatum* sp. HMT204 (Padj = 0.077) (Table S6, Fig. S4a).

Mouthwash users had significantly different population structures to those who did not use mouthwash (Fig. S5, PERMANOVA p = 0.03). MaAsLin2 analysis showed that this difference was associated with reduced levels of the high abundance streptococcal OTU *S. mitis:pneumoniae* (Padj = 0.01) in mouthwash users. *Gr. adiacens* and *R. mucilaginosa* appeared to show increased abundance with mouthwash use, although the adjusted *p* values were not as significant (Fig. S4, Table S6).

## Tooth loss and denture wear and the mucosal microbiome

**Figure 5.**
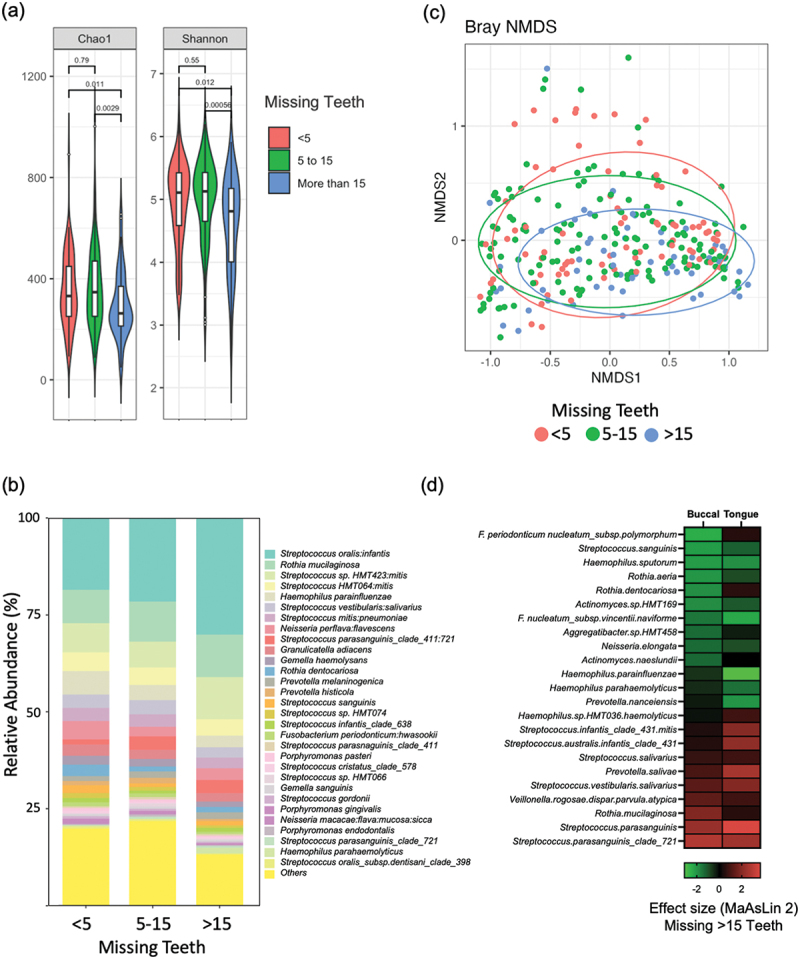
The influence of tooth loss on the mucosal microbiome (a) comparison of Chao1 and Shannon index values in individuals categorised by tooth loss (missing teeth < 5, 5–15 and > 15). (b) Bar plot showing relative abundance (%) of the 30 most abundant species according to number of missing teeth. (c) Community structure analysed by NMDS of Bray-Curtis dissimilarity values according to number of missing teeth. (d) Heatmap comparing the significant changes in species abundance associated with loss of > 15 teeth on buccal and tongue mucosa identified using MaAsLin 2 (fig. S7). Colour intensity corresponds to effect size where reduced abundance (negative values) is indicated in green and increased abundance (positive values) is indicated in red.

The alpha diversities of communities in individuals missing more than 15 teeth were significantly different on all six alpha diversity indices analysed when compared to those with less than five missing teeth (Figure 5a and Fig. S6a). Tooth number had a significant influence on the mucosal microbial population (Figure 5b), with NMDS plots showing clustering of communities from those who had lost more than 15 teeth (PERMANOVA Padj < 0.05; Figure 5c). Missing 5 to 15 and >15 teeth was associated with altered abundance (Padj < 0.1) of 14 and 17 species, respectively (Table S7; Fig. S6b). Missing 5–15 teeth was associated with a reduced abundance of *Haemophilus* spp. and increased abundance of *S. parasanguinis* in addition to many taxa associated with periodontal diseases such as *E. corrodens, P. micra, Saccharibacteria* sp. HMT349, *Fretibacterium* sp. HMT359 and *Treponema* sp. HMT237 (all Padj < 0.1; Table S7). Missing more than 15 teeth was associated with the loss of many taxa reflecting the reduction in richness and biodiversity including *H. parainfluenzae, L. mirabilis, S. sanguinis* and *R. aeria* (all Padj < 0.1; Table S7, Fig. S6).

Due to the highly significant changes associated with tooth loss, we examined if these changes were similar across different mucosal sites. Analysis of buccal and tongue sites identified significant changes at those locations (Fig. S8). Using a heatmap to compare these changes at the buccal and tongue sites, we observed that loss of >15 teeth was associated with decreased abundance of *S. sanguinis* and *H. sputorum* and increased abundance of *S. parasanguinis* at both sites (Figure 5d). However, buccal sites uniquely exhibited increased *R. mucilaginosa* and decreased *R. dentocariosa,* whereas tongue sites exhibited greatly decreased abundance of *H. parainfluenzae* and *H. parahaemolyticus* and increased *S. infantis* (Figure 5d). Analysis of tooth loss in FOM and palate sites did not identify any highly significant results (perhaps influenced by the smaller sample sizes).

Denture wearing also impacted on alpha diversity with reduced biodiversity (Shannon and Simpson) in those with partial or complete dentures (Fig. S8a). Denture wear was associated with significant shifts in population structure (*p* = 0.007; Fig. S8b). We included denture wearing in a multivariate model including smoking and tooth loss to determine if any denture-specific changes could be identified (Fig. S8c). This identified a significant reduction in *S. oralis subsp tigurinus:sanguinis* independent of tooth loss in both partial and complete denture wearers (Padj < 0.04; Figure S8c).

## Impact of age and gender on the mucosal microbiome

Differences in mucosal surface colonisation patterns associated with age and gender were investigated. A multivariate analysis was carried out on age category (<40, 40–60 and >60) and gender including smoking, oral hygiene, brushing frequency and missing teeth (Fig. S9; Table S8). Age over 60 years was associated with a reduced abundance of *Actinomyces* spp., *S. oralis* subsp. *dentasani* and increased abundance of *V. rogosae:dispar*, *Solobacterium moorei* and *Megasphaera micronuciformis* (Fig. S9, Table S8).

Surprisingly, male gender was significantly associated (Padj < 0.05) with increased abundance of several taxa including *Selenomonas sputigena* and *S. parasanguinis* clade 721 (Fig. S9, Table S8). Increased levels of the periodontal disease-associated organisms *T. sockranskii* and *Peptostreptococcaceae* [XI] [G5] *saphenum* were also detected in males, although the *p* values were less significant (Padj 0.11; Fig. S9, Table S8). Although current smoking was significantly associated with male gender (p = 0.047), these changes appeared to be independent of smoking and oral hygiene (Fig. S9).

## Discussion

The oral cavity is one of the most diverse microbiomes in the human body, and this biodiversity is facilitated by the wide range of niches available for microbes to colonise. However, few studies have investigated how these different sites are impacted by different habits, such as smoking and alcohol consumption, oral hygiene practices and the presence/absence of teeth. These studies are important as investigations of the microbiome and its role in mucosal diseases may be confounded by differences associated with these factors when comparing cases and controls. Although our study presents a large group of participants with disparate oral health, smoking and alcohol consumption patterns, it must be noted that most participants (195/256) were sampled while attending a clinic to assess mucosal dysplasia. Sampling at this clinic permitted us to recruit large numbers of current smokers and drinkers which would not have been achievable at general clinics. Although only healthy mucosa were sampled for this study, we cannot exclude that the presence of dysplasia at one site could impact colonisation at other oral sites. To control this, we included ‘clinic attended’ as a variable in our multivariate analysis (Table S3) which demonstrated that this factor did not significantly impact the major findings associated with site, smoking and the other oral health parameters analysed.

There is a general agreement in the literature that the oral microbiome displays site-specific differences and that certain sites can be clustered according to their colonisation patterns, with most agreeing that the dorsum and lateral tongue have similar microbiota while buccal mucosa, palate, FOM and gingival sites also tend to cluster [5,35–39]. In the current study, although oral sites had broadly similar compositions, tongue and palate bacterial communities were generally richer than buccal and FOM samples and this was generally due to increased abundance of the genera *Veillonella, Rothia, Neisseria, Granulicatella* and *Actinomyces* in tongue samples and increased *Prevotella, Veillonella* and *Actinomyces* in palate communities. Previously described streptococcal tropisms could also be identified in the current study, with higher abundance of *S. sanguinis* and *S. mitis* OTUs on buccal mucosa, increased *S. infantis* and *S. parasanguinis* on the tongue and greater abundance of *S. salivarius* and *S. parasanguinis* in palate communities [40]. These species distributions are broadly similar to those described in the literature using a variety of technologies [5,9,40].

However, the high abundance of *Rothia* spp. we noted in this study on the tongue was not seen in some other NGS studies [5,35,37]. However, culture-based studies have shown that the tongue is the main habitat of *R. mucilaginosa* and have estimated relative abundances (~20%) in line with those described here [41]. Tobacco may influence carriage rates of *R. mucilagnosa* on the tongue [42] and the current study contains a high proportion of current smokers (46%) which may have influenced the carriage rates detected here.

Smoking is arguably the most significant influencer of the oral microbiome. The largest study of smoking published to date by Wu et al. sampled saliva and demonstrated that smoking resulted in decreased *Neisseria* and *Haemophilus* and increased *Veillonella* and *Streptococcus* species [17]. Most published studies that have sampled mucosal surfaces have been relatively small and have significantly more non-smokers than smokers in their sample populations, which may potentially influence reported results [24,25]. Our study contained significant numbers of smokers (46%) and our sample size allowed us to analyse the impact in individual mucosal sites. Our findings agreed with many previous studies, including Wu et al. [17], with current smoking being associated with reduced abundance of several species of *Neisseria, H. parainfluenza, L. mirabilis* and *R. aeria*. We also observed reduced *S. australis* and *S. sanguinis* and increased abundance of *S. parasanguinis*. Similar to Wu et al. [17], several Gram-negative bacteria associated with plaque from the genera *Aggregatibacter, Bergeyella, Capnocytophaga, Selenomonas* and *Kingella* also showed significant decreases in abundance in association with smoking (Table S5, **Error! Reference source not found**.). The species showing increased abundance associated with smoking were predominantly subgingival plaque and periodontal disease-associated pathogens, including species from the *Prevotella, Porphyromonas, Tannerella, Parvimonas, Filifactor, Bacteroidales* [G2] and *Peptostreptococcaceae* genera. Wu et al. [17] did not observe any increase in the levels of these periodontal pathogens in their large study of saliva recovered from smokers. However, studies of subgingival plaque have revealed that smoking is associated with increased levels of periodontal pathogens, which agrees with our findings [23]. Either the sampling method of Wu et al. (mouthwash) may not have detected these organisms or the levels of tooth loss and periodontal disease (which were not recorded) may have been very low in their population.

Unexpectedly, some of these smoking-related changes in abundance were found to be highly site-specific. While almost all surfaces examined (buccal, tongue, palate and FOM) exhibited reduced abundance of *Neisseria*, *Lautropia* and *Haemophilus*, depletion of these taxa appears to result in other site-specific shifts in abundance. Buccal sites in smokers showed a highly significant increase in *Prevotella* spp. and generally exhibited the most significant increases in the abundance of putative periodontal pathogens compared to other sites (Fig S2). Increased *Prevotella* was also observed on the palate in some smokers and previous smokers, although this was not statistically significant. This *Prevotella* shift was not evident in FOM samples where smokers exhibited a shift towards *Streptococcus* species. In tongue samples, PCA plots indicated that smokers exhibited decreased levels of *Neisseria*; however, smokers exhibited a divergence in community structure with increased abundance of either *Rothia* or *Streptococcus* species. The heterogeneity of community structure in tongue samples from smokers made it difficult to determine whether these shifts in abundance were statistically significant. It seems on the tongue at least, the response to smoking may be specific to the individual and could be influenced by factors such as the baseline microbiome community structure prior to smoking or other factors not examined here such as diet. For example, individual levels of *Rothia* may be influenced by a diet rich in green leafy vegetables [43].

Very few studies have examined the impact of oral hygiene and periodontal disease on the mucosal microbiome [38]. As subgingival plaque develops and the bacterial population increases, there is a shift to a more anaerobic population dominated by Gram-negative rods and spirochetes [44]. Although we did not measure periodontal disease *per se*, we recorded tooth number and denture wear and assessed plaque levels using the Simplified Greene and Vermillion Index (OHI-S) [26], along with brushing habits and mouthwash use. We found that all of these factors influenced oral mucosal colonisation to some degree. Plaque levels only had a moderate impact on gross community structure as assessed with the Bray-Curtis index but were associated with significantly increased abundance of several periodontitis-associated taxa, including several *Prevotella* species, *Tannerella sp* HMT286, along with plaque-associated species of genera *Leptotrichia, Saccharibacteria* [G1] and *Lachnospiraceae* [G3]. It can take up to 4 days of no oral hygiene for dental plaque to significantly change in structure [44] and as only 5.1% (13 of 256) of our sample population brushed less than once per day, it is perhaps not unexpected that plaque accumulation did not significantly influence the microbiome structure. The impact of moderate plaque accumulation on mucosal surfaces observed here is likely the result of dispersal of plaque associated species onto mucosal sites. In the current study, it is difficult to determine whether this is transient or long-term colonisation and follow-up studies with oral hygiene interventions would be required to determine this.

Surprisingly, brushing frequency appeared to have a more significant impact on mucosal community structure compared to oral hygiene. We found that brushing more than once per day was associated with a reduced abundance of streptococci and an overall increase in biodiversity. We did not ask participants who brushed their teeth regularly whether they also brushed mucosal surfaces. However, the increased biodiversity associated with brushing >1 per day may be associated with frequent rinsing of the oral cavity which may have the effect of reducing the overall abundance of streptococci. Similarly, mouthwash use was associated with reduced levels of some streptococci (most significantly the *S. mitis:pneumonia* OTU) with increased abundance of *G. adiacens* and *R. mucilaginosa*.

Tooth loss was associated with significant changes in alpha and beta diversity. We compared participants who had lost fewer than five teeth with those missing 5–15 teeth or more than 15 teeth. Although the absence of a full periodontal exam is a weakness of the current analysis, we used levels of tooth loss as proxy for periodontal disease status. Both categories (missing 5–15 teeth or more than 15 teeth) exhibited a significant shift in community structure, but only the >15 missing teeth category showed significant changes in richness and biodiversity. This loss in species richness was characterised by reduced abundance of many plaque-associated species including *C. durum*, *L. mirabilis, Haemophilus* spp., *Actinomyces* spp., *Rothia* spp. and *Fusobacterium* spp., presumably related to the loss of tooth surfaces available for colonisation. Those in the 5–15 tooth loss category exhibited microbial signatures indicative of active periodontal disease including increased abundance of *E. corrodens, P. micra, Treponema* sp. HMT237, *Fretibacterium* spp. and *Fusobacterium* spp., presumably due to the greater number of disease active sites shedding pathogens on mucosal surfaces. Socranksy and Huffajee [38] using checkerboard DNA – DNA hybridization also concluded that the soft tissue microbiota differs between healthy subjects and those who have periodontal disease or are edentulous. Their study also showed that mucosal colonisation (tongue and buccal) by *F. nucleatum* and *E. corrodens* was significantly increased in those with periodontitis. Although the current analysis would benefit from periodontal health data, the nature of the microbiome shifts associated with loss of >5 teeth indicate that tooth loss is a reasonable indicator of the presence of periodontal disease.

Interestingly, we also noted site-specific changes in the buccal and tongue communities associated with loss of >15 teeth. On tongue communities, we specifically observed reduced abundance of *H. parainfluenza* and *H. parahaemolyticus* with a concomitant increase in *H. haemolyticus*. Conversely on buccal mucosa, tooth loss was associated with loss of *R. dentocariosa* and increased *R. mucilaginosa*. These site-specific changes may be linked to the baseline microbiomes at these sites. For example, *R. dentocariosa* normally has a higher abundance on buccal mucosa. Tooth loss may result in a decrease in shedding of plaque associated *R. dentocariosa* on buccal mucosa, which may provide space for *R. mucilaginosa* to colonise.

Finally, we also identified some age and gender associated differences in a multivariate analysis with smoking, tooth-loss and other oral hygiene metrics. Loss of *Actinomyces* sp. HMT175 and *S. oralis* subsp *dentisani* was deemed highly significant in those over 60 with an increase in *V. rogosae:dispar*. As these changes are independent of tooth loss, other age-related factors such immunosenescence or dry mouth may be responsible for this. Interestingly, men tended to harbour increased levels of periodontal pathogens *T. sockranskii* and *Peptostreptococcaceae* [XI][G5] *saphenum*. Our multivariate analysis indicated that this was independent of smoking and tooth-loss. The incidence of periodontal disease is known to be higher in males than females and could be related to different abundances of periodontal pathogens. Liu et al. [45] carried out an extensive analysis of gender differences in the oral microbiome and also found several putative periodontal pathogens to have greater abundance in men, including a species of *Treponema*. These changes were linked to hormonal and metabolic differences between the sexes.

We have demonstrated that smoking, tooth loss and oral site are the most significant influencers of the oral mucosal microbiome. The influence of site suggests that niches such as palate, tongue and buccal mucosa should be considered as independent variables in studies of oral mucosal diseases. Our study highlights the need for carefully matched controls in studies examining mucosal colonisation pattern associated with specific pathologies. Due to the difficulties in matching cases and control subjects for these myriad factors, it may be useful to take samples from contralateral healthy mucosal sites (if present) so each patient can act as their own matched control.

## Supplementary Material

Supplemental Material

## References

[1] de Jesus VC, Shikder R, Oryniak D, et al. Sex-based diverse plaque microbiota in children with severe caries. J Dent Res. 2020;99(6):703–13. doi: 10.1177/0022034520908595 32109360

[2] Dzidic M, Collado MC, Abrahamsson T, et al. Oral microbiome development during childhood: an ecological succession influenced by postnatal factors and associated with tooth decay. ISME J. 2018;12(9):2292–2306. doi: 10.1038/s41396-018-0204-z 29899505 PMC6092374

[3] Califf KJ, Schwarzberg-Lipson K, Garg N, et al. Multi-omics analysis of periodontal pocket microbial communities pre- and posttreatment. mSystems. 2017;2(3):e00016–17. doi: 10.1128/mSystems.00016-17 28744486 PMC5513737

[4] Iniesta M, Chamorro C, Ambrosio N, et al. Subgingival microbiome in periodontal health, gingivitis and different stages of periodontitis. J Clin Periodontol. 2023;1(7):905–920. doi: 10.1111/jcpe.13793 36792073

[5] Aas JA, Paster BJ, Stokes LN, et al. Defining the normal bacterial flora of the oral cavity. J Clin Microbiol. 2005;43(11):5721–5732. doi: 10.1128/JCM.43.11.5721-5732.2005 16272510 PMC1287824

[6] Bik EM, Long CD, Armitage GC, et al. Bacterial diversity in the oral cavity of 10 healthy individuals. ISME J. 2010;4(8):962–974. doi: 10.1038/ismej.2010.30 20336157 PMC2941673

[7] Dewhirst FE, Chen T, Izard J, et al. The human oral microbiome. J Bacteriol. 2010;192(19):5002–5017. doi: 10.1128/JB.00542-10 20656903 PMC2944498

[8] Gevers D, Knight R, Petrosino JF, et al. The human microbiome project: a community resource for the healthy human microbiome. PLoS Biol. 2014;10(8):e1001377. doi: 10.1371/journal.pbio.1001377 PMC3419203 22904687

[9] Segata N, Haake SK, Mannon P, et al. Composition of the adult digestive tract bacterial microbiome based on seven mouth surfaces, tonsils, throat and stool samples. Genome Biol. 2012;13(6):R42–R42. doi: 10.1186/gb-2012-13-6-r42 22698087 PMC3446314

[10] Huttenhower C, Gevers D, Knight R, et al. Structure, function and diversity of the healthy human microbiome. Nature. 2014;486:207. 10.1038/nature11234 PMC3564958 22699609

[11] Wade WG, Prosdocimi EM. Profiling of oral bacterial communities. J Dent Res. 2020;99(6):621–629. doi: 10.1177/0022034520914594 32286907 PMC7243418

[12] Escapa IF, Huang Y, Chen T, et al. Construction of habitat-specific training sets to achieve species-level assignment in 16S rRNA gene datasets. Microbiome. 2020;8(1):65. doi: 10.1186/s40168-020-00841-w 32414415 PMC7291764

[13] Eren AM, Borisy GG, Huse SM, et al. Oligotyping analysis of the human oral microbiome. Proc Natl Acad Sci. 2014;111(28):E2875–E2884. doi: 10.1073/pnas.1409644111 24965363 PMC4104879

[14] Crielaard W, Zaura E, Schuller AA, et al. Exploring the oral microbiota of children at various developmental stages of their dentition in the relation to their oral health. BMC Med Genomics. 2011;4(1):22. doi: 10.1186/1755-8794-4-22 21371338 PMC3058002

[15] Mason MR, Chambers S, Dabdoub SM, et al. Characterizing oral microbial communities across dentition states and colonization niches. Microbiome. 2018;6(1):67. doi: 10.1186/s40168-018-0443-2 29631628 PMC5891995

[16] Gomez A, Espinoza JL, Harkins DM, et al. Host Genetic control of the oral microbiome in health and disease. Cell Host Microbe. 2017;22(3):269–278.e3. doi: 10.1016/j.chom.2017.08.013 28910633 PMC5733791

[17] Wu J, Peters BA, Dominianni C, et al. Cigarette smoking and the oral microbiome in a large study of American adults. ISME J. 2016;10(10):2435–2446. doi: 10.1038/ismej.2016.37 27015003 PMC5030690

[18] Nearing JT, DeClercq V, Limbergen JV, et al. Assessing the variation within the oral microbiome of healthy adults. mSphere. 2020;5(5): doi: 10.1128/mSphere.00451-20 PMC7529435 32999079

[19] Marsh PD. Microbial ecology of dental plaque and its significance in health and disease. Adv Dent Res. 1994;8(2):263–271. doi: 10.1177/08959374940080022001 7865085

[20] Griffen AL, Beall CJ, Campbell JH, et al. Distinct and complex bacterial profiles in human periodontitis and health revealed by 16S pyrosequencing. ISME J. 2011;6(6):1176–1185. doi: 10.1038/ismej.2011.191 22170420 PMC3358035

[21] Socransky SS, Haffajee AD, Cugini MA, et al. Microbial complexes in subgingival plaque. J Clin Periodontol. 2005;25(2):134–144. doi: 10.1111/j.1600-051X.1998.tb02419.x 9495612

[22] Hsiao J-R, Chang C-C, Lee W-T, et al. The interplay between oral microbiome, lifestyle factors and genetic polymorphisms in the risk of oral squamous cell carcinoma. Carcinogenesis. 2018;39(6):778–787. doi: 10.1093/carcin/bgy053 29668903

[23] Moon JH, Lee JH, Lee JY. Subgingival microbiome in smokers and non‐smokers in Korean chronic periodontitis patients. Mol Oral Microbiol. 2015;30:227–241. doi: 10.1111/omi.12086 25283067

[24] Charlson ES, Chen J, Custers-Allen R, et al. Disordered microbial communities in the upper respiratory tract of cigarette smokers. PLoS One. 2010;5(12):e15216. doi: 10.1371/journal.pone.0015216 21188149 PMC3004851

[25] Thomas AM, Gleber-Netto FO, Fernandes GR, et al. Alcohol and tobacco consumption affects bacterial richness in oral cavity mucosa biofilms. BMC Microbiol. 2014;14(1):250. doi: 10.1186/s12866-014-0250-2 25278091 PMC4186948

[26] Greene JG, Vermillion JR. The simplified oral hygiene index. J Am Dent Assoc. 1964;68(1):7–13. doi: 10.14219/jada.archive.1964.0034 14076341

[27] Amer A, Galvin S, Healy CM, et al. The microbiome of potentially malignant oral leukoplakia exhibits enrichment for *Fusobacterium, Leptotrichia, Campylobacter*, and *Rothia* species. Front Microbiol. 2017;8:2391. doi: 10.3389/fmicb.2017.02391 29250055 PMC5717034

[28] Frank JA, Reich CI, Sharma S, et al. Critical evaluation of two primers commonly used for Amplification of bacterial 16S rRNA genes. Appl Environ Microb. 2008;74(8):2461–2470. doi: 10.1128/AEM.02272-07 PMC2293150 18296538

[29] Comeau AM, Douglas GM, Langille MGI, et al. Microbiome helper: a custom and streamlined workflow for microbiome Research. mSystems. 2017;2(1):e00127–16. doi: 10.1128/mSystems.00127-16 PMC5209531 28066818

[30] Callahan BJ, McMurdie PJ, Rosen MJ, et al. DADA2: high-resolution sample inference from Illumina amplicon data. Nat Methods. 2016;13(7):581–583. doi: 10.1038/nmeth.3869 27214047 PMC4927377

[31] McMurdie PJ, Holmes S, Watson M. Phyloseq: an R package for reproducible interactive analysis and graphics of microbiome census data. PLoS One. 2013;8(4):e61217. doi: 10.1371/journal.pone.0061217 23630581 PMC3632530

[32] Xu S, Li Z, Tang W, et al. MicrobiotaProcess: a comprehensive R package for managing and analyzing microbiome and other ecological data within the tidy framework. Innovation (Camb). 2023;4:100388. 36895758 10.1016/j.xinn.2023.100388 PMC9988672

[33] Barnett D, Arts I, Penders J. microViz: an R package for microbiome data visualization and statistics. J Open Source Softw. 2021;6(63):3201. doi: 10.21105/joss.03201

[34] Mallick H, Rahnavard A, McIver LJ, et al. Multivariable association discovery in population-scale meta-omics studies. PLoS Comput Biol. 2021;17(11):e1009442. doi: 10.1371/journal.pcbi.1009442 34784344 PMC8714082

[35] Mager DL, Ximenez‐Fyvie LA, Haffajee AD, et al. Distribution of selected bacterial species on intraoral surfaces. J Clin Periodontol. 2003;30(7):644–654. doi: 10.1034/j.1600-051X.2003.00376.x 12834503

[36] Welch JLM, Dewhirst FE, Borisy GG. Biogeography of the oral microbiome: the site-specialist hypothesis. Annu Rev Microbiol. 2019;73(1):335–358. doi: 10.1146/annurev-micro-090817-062503 31180804 PMC7153577

[37] Segata N, Haake SK, Mannon P, et al. Composition of the adult digestive tract bacterial microbiome based on seven mouth surfaces, tonsils, throat and stool samples. Genome Biol. 2012;13(6):R42. doi: 10.1186/gb-2012-13-6-r42 22698087 PMC3446314

[38] Socransky SS, Huffajee AD. Periodontal microbial ecology. Periodontol 2000. 2005;38(1):135–187. doi: 10.1111/j.1600-0757.2005.00107.x 15853940

[39] Diaz PI, Dupuy AK, Abusleme L, et al. Using high throughput sequencing to explore the biodiversity in oral bacterial communities. Mol Oral Microbiol. 2012;27(3):182–201. doi: 10.1111/j.2041-1014.2012.00642.x 22520388 PMC3789374

[40] McLean AR, Torres‐Morales J, Dewhirst FE, et al. Site‐tropism of streptococci in the oral microbiome. Mol Oral Microbiol. 2022;37(6):229–243. doi: 10.1111/omi.12387 36073311 PMC9691528

[41] Uchibori S, Tsuzukibashi O, Kobayashi T, et al. Localization of the genus *Rothia* in the oral cavity. Int J Oral-Medical Sci. 2013;11(3):207–210. doi: 10.5466/ijoms.11.207

[42] Halboub E, Al-Ak’hali M S, Alamir A H, Homeida H E, Baraniya D, Chen T and Al-Hebshi N Noor. (2020). Tongue microbiome of smokeless tobacco users. BMC Microbiol, 20(1), doi: 10.1186/s12866-020-01883-8 PMC7346439 32640977

[43] Velmurugan S, Gan JM, Rathod KS, et al. Dietary nitrate improves vascular function in patients with hypercholesterolemia: a randomized, double-blind, placebo-controlled study. Am J Clin Nutrition. 2015;103(1):25–38. doi: 10.3945/ajcn.115.116244 26607938 PMC4691670

[44] Teughels W, Christof G, Quirynen M, Jakubovics, N Biofilm and Periodontal Microbiology. Carranza’s Clinical Periodontology. Elsevier Health Sciences. 2012; 132–169.

[45] Liu X, Tong X, Jie Z, et al. Sex differences in the oral microbiome, host traits, and their causal relationships. iScience. 2023;26(1):105839. doi: 10.1016/j.isci.2022.105839 36660475 PMC9843272

