# Supplementary material for: Smoking, tooth loss and oral hygiene practices have significant and site-specific impacts on the microbiome of oral mucosal surfaces: a cross-sectional study
Source: J Oral Microbiol. 2023 Oct 2;15(1):2263971. doi: 10.1080/20002297.2023.2263971 (PMC10547447; doi:10.1080/20002297.2023.2263971)
Supplement: Supplemental Material [file ZJOM_A_2263971_SM6514.docx]

**Supplementary data for:**

# **Smoking, tooth loss and oral hygiene practices have significant and site-specific impacts on the microbiome of oral mucosal surfaces**

**Submitted to the Journal of Oral Microbiology**

**Table S1.** Participant demographics and comparison of variables associated with oral medicine and general dental patients.

| **Demographic** | **Oral Medicine Clinic** | **General Dental Clinic** | **p value** |
| --- | --- | --- | --- |
| Total | 195 | 61 | - |
|  |  |  |  |
| **Sites** |  |  |  |
| Floor of mouth | 26 | 17 |  |
| Buccal mucosa | 84 | 43 |  |
| Tongue | 73 | 43 |  |
| Palate | 19 | 17 | 0.52 |
|  |  |  |  |
| **Gender** |  |  |  |
| Males:Females | 102:93 | 21:40 | 0.015 |
|  |  |  |  |
| **Age range** |  |  |  |
| <40 | 18 | 17 |  |
| 40-60 | 91 | 30 |  |
| >60 | 86 | 13 |  |
| ND | 0 | 1 | 0.0001 |
|  |  |  |  |
| **Smoking status** |  |  |  |
| Current smoker | 103 | 15 |  |
| Former smoker | 55 | 15 |  |
| Never smoker | 37 | 31 | 0.000002 |
|  |  |  |  |
| **Alcohol units/week** |  |  |  |
| 0 | 41 | 13 |  |
| 1 to 10 | 99 | 41 |  |
| 11 to 20 | 26 | 2 |  |
| More than 20 | 29 | 5 | 0.03 |
|  |  |  |  |
| **Alcohol >20 units/week and smoker** |  |  |  |
| Yes | 30 | 1 |  |
| No | 165 | 60 | 0.0023 |
|  |  |  |  |
| **Missing teeth** |  |  |  |
| <5 | 25 | 26 |  |
| 5 to 15 | 80 | 26 |  |
| More than 15 | 40 | 7 |  |
| ND | 50 | 2 | 0.0002 |
|  |  |  |  |
| **OHI Score** |  |  |  |
| Good (OHI-S < 0.5) | 40 | 37 |  |
| Fair (OHI-S 0.5-1) | 61 | 15 |  |
| Poor (OHI-S > 1) | 40 | 7 |  |
| Edentulous | 9 | 0 |  |
| Not recorded | 45 | 2 | 0.00003 |
|  |  |  |  |
| **Brushing times/day** |  |  |  |
| <1 | 13 | 0 |  |
| 1 | 29 | 5 |  |
| >1 | 130 | 56 |  |
| Not recorded | 23 | 0 | 0.009 |
|  |  |  |  |
| **Denture** |  |  |  |
| Complete (F/F) | 10 | 1 |  |
| Partial | 38 | 7 |  |
| None | 147 | 53 | 0.201 |
|  |  |  |  |
| **Mouthwash use** |  |  |  |
| No | 26 | 106 |  |
| Yes | 89 | 35 | 0.109 |


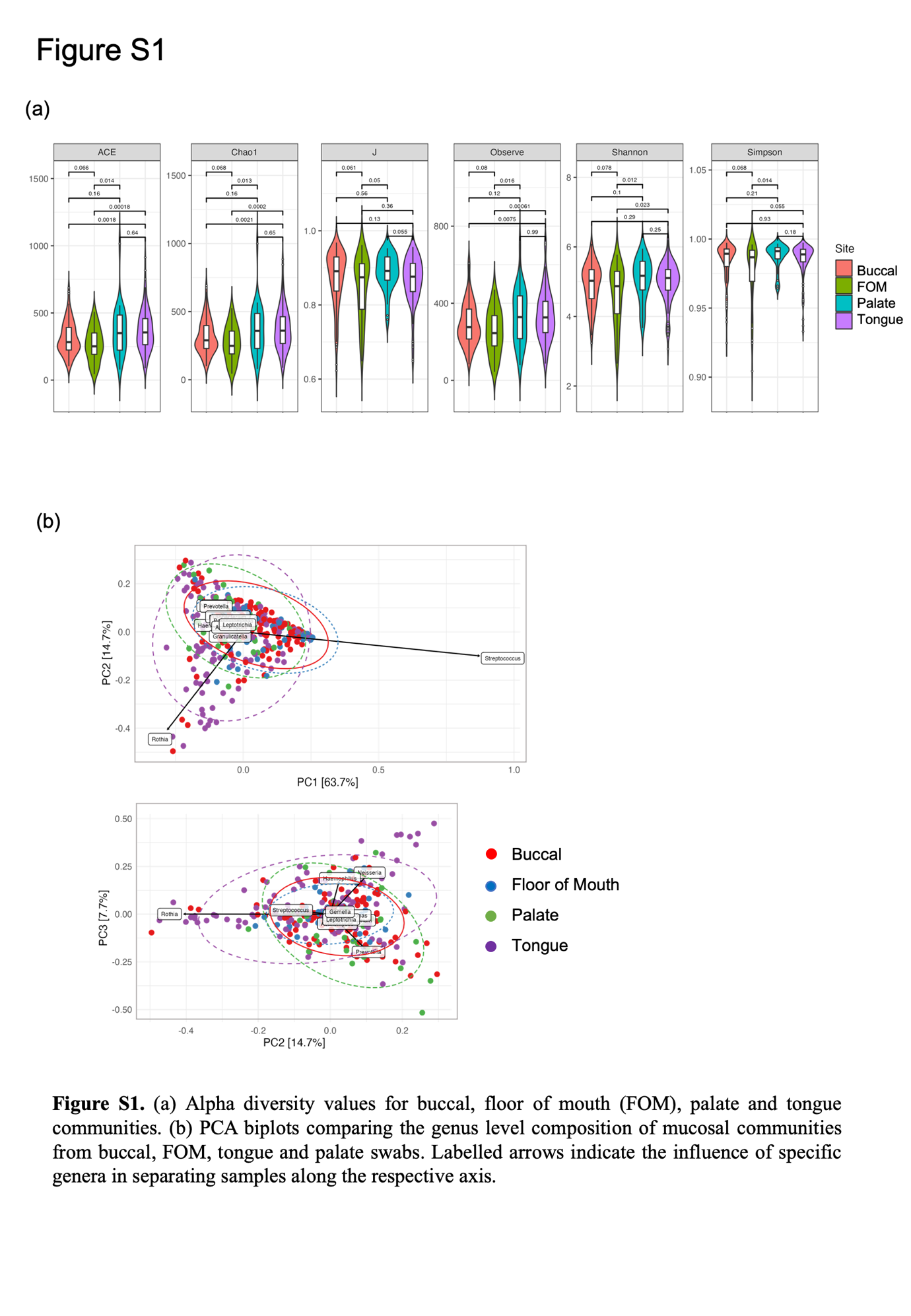


**Table S2.** Results of MaAsLin2 analysis showing species with significant changes in abundance (Padj<0.05) associated with site.

| **Species** | **Site** | **Coefficient** | **P value** | **Padj** |
| --- | --- | --- | --- | --- |
| *Rothia aeria dentocariosa* | Tongue | 0.84 | 6.52E-12 | 3.66E-09 |
| *Veillonella rogosae dispar* | Tongue | 0.85 | 2.28E-09 | 4.27E-07 |
| *Rothia mucilaginosa* | Tongue | 0.51 | 4.56E-09 | 6.40E-07 |
| *Granulicatella adiacens* | Tongue | 0.39 | 1.71E-06 | 0.0002 |
| *Veillonella rogosae dispar parvula atypica* | Tongue | 0.38 | 3.42E-06 | 0.0003 |
| *Veillonella denticariosi* | Tongue | 0.51 | 1.17E-05 | 0.0006 |
| *Streptococcus* sp*. HMT061* | Tongue | 0.48 | 1.82E-05 | 0.0008 |
| *Actinomyces* sp. *HMT180 odontolyticus* | Tongue | 0.51 | 2.89E-05 | 0.001 |
| *Streptococcus parasanguinis clade 411:721* | Tongue | 0.58 | 7.01E-05 | 0.002 |
| *Streptococcus* sp. *HMT423 mitis* | Tongue | -0.51 | 8.09E-05 | 0.002 |
| *Gemella haemolysans* | Tongue | -0.63 | 0.0001 | 0.003 |
| *Actinomyces oris* | Tongue | -0.27 | 0.0001 | 0.003 |
| *Streptococcus mitis pneumoniae* | Tongue | -0.71 | 0.0002 | 0.003 |
| *Streptococcus* sp. *HMT064 mitis* | Tongue | -0.51 | 0.0002 | 0.003 |
| *Streptococcus parasanguinis clade 411* | Tongue | 0.54 | 0.0002 | 0.004 |
| *Streptococcus infantis clade 638* | Tongue | 0.48 | 0.0003 | 0.006 |
| *Actinomyces lingnae* | Tongue | 0.38 | 0.001 | 0.01 |
| *Gemella sanguinis* | Tongue | 0.43 | 0.001 | 0.01 |
| *Streptococcus thermophilus salivarius* | Tongue | 0.21 | 0.001 | 0.01 |
| *Streptococcus sanguinis* | Tongue | -0.37 | 0.001 | 0.02 |
| *Actinomyces odontolyticus* | Tongue | 0.38 | 0.002 | 0.02 |
| *Streptococcus australis* | Tongue | 0.38 | 0.002 | 0.02 |
| *Haemophilus pittmaniae* | Tongue | 0.16 | 0.003 | 0.03 |
| *Streptococcus oralis* subsp. *tigurinus clade 070* | Tongue | -0.34 | 0.003 | 0.04 |
| *Prevotella* sp. *HMT300* | Tongue | -0.19 | 0.003 | 0.04 |
| *Parvimonas micra* | Tongue | -0.37 | 0.004 | 0.04 |
| *Mogibacterium timidum* | Tongue | -0.25 | 0.004 | 0.04 |
| *Corynebacterium matruchotii* | Tongue | -0.27 | 0.005 | 0.045 |
| *Streptococcus constellatus intermedius* | FOM | -0.54 | 0.004 | 0.04 |
| *Prevotella sp. HMT305* | Palate | 0.56 | 3.74E-10 | 1.05E-07 |
| *Peptostreptococcaceae XI G 1 sulci* | Palate | 0.41 | 2.08E-07 | 2.34E-05 |
| *Mogibacterium diversum* | Palate | 0.65 | 4.43E-06 | 0.0003 |
| *Saccharibacteria TM7 G 1 bacterium HMT352* | Palate | 0.55 | 6.49E-06 | 0.0004 |
| *Prevotella pallens* | Palate | 0.72 | 9.99E-06 | 0.0006 |
| *Prevotella salivae* | Palate | 0.96 | 1.38E-05 | 0.0006 |
| *Prevotella histicola* | Palate | 1.05 | 2.00E-05 | 0.0008 |
| *Selenomonas* sp. *HMT136* | Palate | 0.40 | 2.84E-05 | 0.001 |
| *Atopobium parvulum* | Palate | 0.82 | 5.94E-05 | 0.002 |
| *Alloprevotella* sp*. HMT308* | Palate | 0.66 | 5.78E-05 | 0.002 |
| *Solobacterium moorei* | Palate | 0.62 | 5.48E-05 | 0.002 |
| *Streptococcus salivarius* | Palate | 0.42 | 6.38E-05 | 0.002 |
| *Streptococcus vestibularis thermophilus salivarius* | Palate | 0.47 | 0.0001 | 0.003 |
| *Lachnoanaerobaculum orale* | Palate | 0.34 | 0.0002 | 0.005 |
| *Stomatobaculum sp. HMT097* | Palate | 0.42 | 0.001 | 0.009 |
| *Rothia dentocariosa* | Palate | -0.62 | 0.001 | 0.009 |
| *Streptococcus parasanguinis clade 721* | Palate | 0.68 | 0.001 | 0.01 |
| *Actinomyces* sp*. HMT172* | Palate | 0.60 | 0.001 | 0.01 |
| *Prevotella* sp*. HMT313* | Palate | 0.65 | 0.001 | 0.01 |
| *Actinomyces odontolyticus* | Palate | 0.62 | 0.001 | 0.01 |
| *Haemophilus haemolyticus* | Palate | -0.45 | 0.001 | 0.01 |
| *Actinomyces graevenitzii* | Palate | 0.69 | 0.001 | 0.01 |
| *Parvimonas micra* | Palate | -0.62 | 0.001 | 0.02 |
| *Fusobacterium nucleatum_subsp vincentii* | Palate | -0.56 | 0.002 | 0.02 |
| *Actinomyces lingnae* | Palate | 0.49 | 0.002 | 0.03 |
| *Leptotrichia* sp*. HMT215* | Palate | 0.45 | 0.003 | 0.04 |
| *Leptotrichia hongkongensis* | Palate | -0.37 | 0.003 | 0.04 |
| *Streptococcus* sp*. HMT057* | Palate | 0.56 | 0.004 | 0.04 |
| *Campylobacter concisus* | Palate | 0.46 | 0.004 | 0.04 |
| *Prevotella melaninogenica* | Palate | 0.55 | 0.005 | 0.045 |

**Table S3.** Results of a multivariate MaAsLin2 analysis showing species exhibiting significant changes in abundance where mucosal site, smoking, oral hygiene and tooth loss are included in the model as fixed effects and clinic attended (oral medicine or general dentistry clinic) is included as a random effect.

| **Species** | **Variable** | **Coefficient** | **P val** | **P adj** |
| --- | --- | --- | --- | --- |
| *Rothia aeria dentocariosa* | Tongue | 2.36 | 0.000 | 0.000 |
| *Rothia mucilaginosa* | Tongue | 1.44 | 0.000 | 0.002 |
| *Granulicatella adiacens* | Tongue | 1.11 | 0.000 | 0.011 |
| *Streptococcus infantis_clade_638* | Tongue | 1.84 | 0.000 | 0.014 |
| *Actinomyces oris* | Tongue | -1.06 | 0.000 | 0.018 |
| *Veillonella rogosae dispar* | Tongue | 1.93 | 0.001 | 0.022 |
| *Streptococcus parasanguinis_clade_411_721* | Tongue | 1.83 | 0.001 | 0.029 |
| *Streptococcus sp HMT064 mitis* | Tongue | -1.62 | 0.001 | 0.034 |
| *Streptococcus sp HMT061* | Tongue | 1.39 | 0.001 | 0.034 |
| *Streptococcus oralis_subsp tigurinus_clade_070* | Tongue | -1.38 | 0.001 | 0.039 |
| *Veillonella rogosae dispar parvula atypica* | Tongue | 0.98 | 0.002 | 0.050 |
| *Rothia dentocariosa* | Tongue | -1.35 | 0.003 | 0.069 |
| *Corynebacterium matruchotii* | Tongue | -1.11 | 0.004 | 0.079 |
| *Streptococcus sp HMT423 mitis* | Tongue | -1.44 | 0.004 | 0.090 |
| *Streptococcus sanguinis* | Tongue | -1.23 | 0.005 | 0.091 |
| *Actinomyces sp HMT169* | Tongue | -1.25 | 0.006 | 0.104 |
| *Actinomyces sp HMT180 odontolyticus* | Tongue | 1.34 | 0.008 | 0.119 |
| *Gemella sanguinis* | Tongue | 1.31 | 0.010 | 0.147 |
| *Streptococcus oralis_subsp tigurinus_clade_071* | Tongue | -1.13 | 0.010 | 0.147 |
| *Eikenella corrodens* | Tongue | -0.83 | 0.013 | 0.166 |
| *Streptococcus mitis pneumoniae* | Tongue | -1.85 | 0.014 | 0.168 |
| *Parvimonas micra* | Tongue | -1.22 | 0.015 | 0.177 |
| *Actinomyces sp HMT175* | Tongue | -1.06 | 0.016 | 0.178 |
| *Streptococcus australis* | Tongue | 1.12 | 0.017 | 0.186 |
| *Fusobacterium nucleatum_subsp vincentii naviforme* | Tongue | -1.22 | 0.018 | 0.197 |
| *Peptostreptococcaceae XI G 4 bacterium_HMT369* | Tongue | -0.73 | 0.020 | 0.200 |
| *Capnocytophaga granulosa* | Tongue | -0.86 | 0.019 | 0.200 |
| *Prevotella sp HMT305* | Palate | 2.44 | 0.000 | 0.000 |
| *Peptostreptococcaceae XI G 1 sulci* | Palate | 1.73 | 0.000 | 0.000 |
| *Prevotella pallens* | Palate | 3.37 | 0.000 | 0.000 |
| *Prevotella salivae* | Palate | 4.38 | 0.000 | 0.000 |
| *Streptococcus salivarius* | Palate | 2.23 | 0.000 | 0.000 |
| *Saccharibacteria TM7 G 1 bacterium_HMT352* | Palate | 2.40 | 0.000 | 0.000 |
| *Mogibacterium diversum* | Palate | 2.58 | 0.000 | 0.000 |
| *Streptococcus vestibularis thermophilus salivarius* | Palate | 2.44 | 0.000 | 0.000 |
| *Streptococcus parasanguinis_clade_721* | Palate | 3.57 | 0.000 | 0.000 |
| *Atopobium parvulum* | Palate | 3.46 | 0.000 | 0.001 |
| *Prevotella histicola* | Palate | 4.06 | 0.000 | 0.001 |
| *Solobacterium moorei* | Palate | 2.51 | 0.000 | 0.003 |
| *Lachnoanaerobaculum orale* | Palate | 1.43 | 0.000 | 0.005 |
| *Actinomyces odontolyticus* | Palate | 2.85 | 0.000 | 0.006 |
| *Prevotella sp HMT313* | Palate | 2.86 | 0.000 | 0.006 |
| *Selenomonas sp HMT136* | Palate | 1.52 | 0.000 | 0.006 |
| *Actinomyces graevenitzii* | Palate | 3.12 | 0.000 | 0.008 |
| *Alloprevotella sp HMT308* | Palate | 2.45 | 0.000 | 0.009 |
| *Leptotrichia sp HMT417* | Palate | 3.25 | 0.000 | 0.010 |
| *Prevotella melaninogenica* | Palate | 2.87 | 0.000 | 0.011 |
| *Actinomyces lingnae* | Palate | 2.20 | 0.000 | 0.014 |
| *Campylobacter concisus* | Palate | 2.26 | 0.000 | 0.015 |
| *Actinomyces sp HMT172* | Palate | 2.49 | 0.000 | 0.015 |
| *Streptococcus sp HMT057* | Palate | 2.49 | 0.001 | 0.023 |
| *Eikenella corrodens* | Palate | -1.63 | 0.001 | 0.031 |
| *Parvimonas micra* | Palate | -2.44 | 0.001 | 0.033 |
| *Oribacterium sinus* | Palate | 2.19 | 0.002 | 0.040 |
| *Streptococcus parasanguinis_clade_411_721* | Palate | 2.57 | 0.002 | 0.042 |
| *Rothia mucilaginosa* | Palate | 1.52 | 0.002 | 0.044 |
| *Megasphaera micronuciformis* | Palate | 1.27 | 0.002 | 0.050 |
| *Leptotrichia hongkongensis* | Palate | -1.61 | 0.003 | 0.070 |
| *Saccharibacteria TM7 G 3 bacterium_HMT351* | Palate | 1.63 | 0.005 | 0.091 |
| *Streptococcus vestibularis salivarius* | Palate | 1.85 | 0.005 | 0.095 |
| *Fusobacterium nucleatum_subsp vincentii* | Palate | -2.10 | 0.006 | 0.104 |
| *Aggregatibacter sp HMT458* | Palate | -1.17 | 0.006 | 0.104 |
| *Tannerella forsythia* | Palate | -1.34 | 0.006 | 0.104 |
| *Haemophilus haemolyticus* | Palate | -1.40 | 0.011 | 0.158 |
| *Porphyromonas catoniae* | Palate | -0.67 | 0.012 | 0.163 |
| *Treponema socranskii* | Palate | -1.01 | 0.013 | 0.164 |
| *Stomatobaculum longum* | Palate | 1.17 | 0.014 | 0.169 |
| *Haemophilus sputorum* | Palate | -1.20 | 0.015 | 0.177 |
| *Prevotella nanceiensis* | Palate | 1.21 | 0.017 | 0.191 |
| *Actinomyces sp HMT180* | Palate | 0.81 | 0.018 | 0.196 |
| *Actinomyces sp HMT172 sp HMT180 odontolyticus* | Palate | 1.52 | 0.019 | 0.200 |
| *Veillonella sp HMT780* | Palate | -0.84 | 0.019 | 0.200 |
| *Leptotrichia wadei* | Palate | -2.01 | 0.021 | 0.205 |
| *Streptococcus infantis_clade_431* | FOM | -1.63 | 0.006 | 0.103 |
| *Parvimonas micra* | FOM | -1.58 | 0.016 | 0.183 |
| *Prevotella pleuritidis* | Current smoker | 2.14 | 0.000 | 0.000 |
| *Lautropia mirabilis* | Current smoker | -2.19 | 0.000 | 0.001 |
| *Neisseria perflava flavescens* | Current smoker | -3.18 | 0.000 | 0.003 |
| *Kingella oralis* | Current smoker | -2.31 | 0.000 | 0.004 |
| *Stomatobaculum sp HMT097* | Current smoker | -1.44 | 0.000 | 0.006 |
| *Clostridiales F 1 G 1 bacterium_HMT093* | Current smoker | 0.92 | 0.000 | 0.006 |
| *Abiotrophia defectiva* | Current smoker | -1.17 | 0.000 | 0.006 |
| *Haemophilus parainfluenzae* | Current smoker | -1.84 | 0.000 | 0.006 |
| *Peptostreptococcaceae XI G 5 saphenum* | Current smoker | 1.61 | 0.000 | 0.014 |
| *Streptococcus intermedius* | Current smoker | -1.37 | 0.000 | 0.015 |
| *Streptococcus sp HMT066* | Current smoker | -2.07 | 0.000 | 0.016 |
| *Selenomonas artemidis* | Current smoker | -1.32 | 0.001 | 0.022 |
| *Cardiobacterium hominis* | Current smoker | -1.01 | 0.001 | 0.028 |
| *Streptococcus sp HMT066 sp HMT074* | Current smoker | -1.93 | 0.001 | 0.028 |
| *Prevotella histicola* | Current smoker | 2.11 | 0.002 | 0.040 |
| *Bacteroidales G 2 bacterium_HMT274* | Current smoker | 1.21 | 0.003 | 0.055 |
| *Streptococcus australis* | Current smoker | -1.48 | 0.003 | 0.065 |
| *Gemella morbillorum* | Current smoker | -1.39 | 0.004 | 0.080 |
| *Bergeyella sp HMT322* | Current smoker | -0.99 | 0.005 | 0.090 |
| *Aggregatibacter sp HMT512* | Current smoker | -0.85 | 0.005 | 0.091 |
| *Streptococcus parasanguinis_clade_411_721* | Current smoker | 1.61 | 0.006 | 0.104 |
| *Streptococcus oralis_subsp tigurinus_clade_071* | Current smoker | 1.28 | 0.006 | 0.104 |
| *Prevotella sp HMT313* | Current smoker | 1.43 | 0.006 | 0.104 |
| *Mogibacterium timidum* | Current smoker | 0.99 | 0.006 | 0.104 |
| *Fusobacterium periodonticum hwasookii* | Current smoker | -1.35 | 0.008 | 0.119 |
| *Actinomyces sp HMT169* | Current smoker | -1.30 | 0.008 | 0.119 |
| *Actinomyces lingnae* | Current smoker | 1.16 | 0.008 | 0.119 |
| *Rothia aeria* | Current smoker | -1.30 | 0.008 | 0.126 |
| *Haemophilus sp HMT036 haemolyticus sp HMT908* | Current smoker | 1.10 | 0.010 | 0.142 |
| *Filifactor alocis* | Current smoker | 1.38 | 0.012 | 0.160 |
| *Streptococcus sp HMT064 sp HMT423 mitis* | Current smoker | 0.81 | 0.012 | 0.163 |
| *Stomatobaculum longum* | Current smoker | 0.87 | 0.012 | 0.164 |
| *Mycoplasma faucium* | Current smoker | 0.88 | 0.012 | 0.164 |
| *Actinomyces graevenitzii* | Current smoker | 1.45 | 0.013 | 0.166 |
| *Atopobium parvulum* | Current smoker | 1.34 | 0.013 | 0.167 |
| *Aggregatibacter sp HMT458* | Current smoker | -0.75 | 0.013 | 0.167 |
| *Tannerella forsythia* | Current smoker | 0.87 | 0.014 | 0.169 |
| *Streptococcus sanguinis* | Current smoker | -1.14 | 0.015 | 0.177 |
| *Porphyromonas catoniae* | Current smoker | -0.47 | 0.015 | 0.177 |
| *Streptococcus intermedius* | Former smoker | -1.84 | 0.000 | 0.001 |
| *Selenomonas artemidis* | Former smoker | -1.57 | 0.000 | 0.008 |
| *Streptococcus gordonii* | Former smoker | -2.62 | 0.000 | 0.013 |
| *Streptococcus sp HMT057* | Former smoker | 1.65 | 0.003 | 0.070 |
| *Campylobacter gracilis* | Former smoker | -1.39 | 0.006 | 0.101 |
| *Streptococcus oralis_subsp dentisani_clade_398* | Former smoker | -1.00 | 0.007 | 0.109 |
| *Saccharibacteria TM7 G 1 bacterium_HMT349* | Former smoker | -0.84 | 0.010 | 0.147 |
| *Streptococcus australis oralis_subsp dentisani* | Former smoker | 0.92 | 0.011 | 0.148 |
| *Actinomyces sp HMT169* | Former smoker | -1.29 | 0.013 | 0.166 |
| *Actinomyces sp HMT448* | Former smoker | -1.06 | 0.013 | 0.166 |
| *Ruminococcaceae G 2 bacterium_HMT085* | Former smoker | 0.81 | 0.014 | 0.169 |
| *Cardiobacterium hominis* | Former smoker | -0.78 | 0.015 | 0.177 |
| *Gemella haemolysans* | Former smoker | 1.72 | 0.019 | 0.200 |
| *Streptococcus constellatus intermedius* | Missing 5-15 teeth | 2.17 | 0.000 | 0.002 |
| *Eikenella corrodens* | Missing 5-15 teeth | 1.16 | 0.000 | 0.015 |
| *Saccharibacteria TM7 G 1 bacterium_HMT349* | Missing 5-15 teeth | 0.97 | 0.000 | 0.016 |
| *Haemophilus sputorum* | Missing 5-15 teeth | -1.08 | 0.001 | 0.028 |
| *Streptococcus parasanguinis_clade_721* | Missing 5-15 teeth | 1.61 | 0.001 | 0.031 |
| *Haemophilus haemolyticus* | Missing 5-15 teeth | -1.14 | 0.002 | 0.040 |
| *Streptococcus parasanguinis_clade_411 _721* | Missing 5-15 teeth | 1.65 | 0.002 | 0.043 |
| *Campylobacter gracilis* | Missing 5-15 teeth | 1.30 | 0.002 | 0.046 |
| *Parvimonas micra* | Missing 5-15 teeth | 1.29 | 0.008 | 0.119 |
| *Streptococcus intermedius* | Missing 5-15 teeth | 0.89 | 0.009 | 0.126 |
| *Peptostreptococcaceae XI G 9 brachy* | Missing 5-15 teeth | 1.10 | 0.011 | 0.148 |
| *Fretibacterium sp HMT359* | Missing 5-15 teeth | 0.64 | 0.016 | 0.182 |
| *Streptococcus cristatus_clade_578* | Missing 5-15 teeth | -1.04 | 0.019 | 0.200 |
| *Streptococcus australis* | Missing 5-15 teeth | -1.05 | 0.019 | 0.200 |
| *Haemophilus sp HMT036 haemolyticus sp HMT908* | Missing 5-15 teeth | -0.89 | 0.019 | 0.200 |
| *Haemophilus sputorum* | Missing >15 teeth | -1.70 | 0.001 | 0.025 |
| *Streptococcus salivarius* | Missing >15 teeth | 1.47 | 0.001 | 0.028 |
| *Leptotrichia sp HMT417* | Missing >15 teeth | 2.45 | 0.005 | 0.092 |
| *Haemophilus parainfluenzae* | Missing >15 teeth | -1.79 | 0.007 | 0.110 |
| *Streptococcus vestibularis thermophilus salivarius* | Missing >15 teeth | 1.26 | 0.013 | 0.164 |
| *Actinomyces lingnae* | Missing >15 teeth | -1.48 | 0.015 | 0.177 |
| *Streptococcus parasanguinis_clade_411_721* | Missing >15 teeth | 1.93 | 0.019 | 0.200 |
| *Campylobacter rectus* | Oral hygiene Fair | -0.60 | 0.003 | 0.055 |
| *Haemophilus parahaemolyticus* | Oral hygiene Fair | -0.76 | 0.005 | 0.091 |
| *Streptococcus salivarius* | Oral hygiene Fair | -0.82 | 0.006 | 0.105 |
| *Streptococcus gordonii* | Oral hygiene Fair | 1.73 | 0.007 | 0.117 |
| *Streptococcus cristatus_clade_578* | Oral hygiene Fair | 1.26 | 0.008 | 0.119 |
| *Kingella oralis* | Oral hygiene Fair | -1.25 | 0.022 | 0.208 |
| *Prevotella denticola* | Oral hygiene Poor | 1.96 | 0.001 | 0.022 |
| *Lachnospiraceae G 3 bacterium_HMT100* | Oral hygiene Poor | 1.48 | 0.001 | 0.039 |
| *Prevotella sp HMT313* | Oral hygiene Poor | 1.90 | 0.001 | 0.039 |
| *Leptotrichia sp HMT498* | Oral hygiene Poor | 1.41 | 0.002 | 0.042 |
| *Fusobacterium sp HMT204* | Oral hygiene Poor | 1.28 | 0.002 | 0.048 |
| *Streptococcus sp HMT057* | Oral hygiene Poor | 1.82 | 0.002 | 0.050 |
| *Saccharibacteria TM7 G 1 bacterium_HMT346* | Oral hygiene Poor | 1.00 | 0.007 | 0.118 |
| *Tannerella sp HMT286* | Oral hygiene Poor | 1.26 | 0.009 | 0.126 |
| *Lachnoanaerobaculum saburreum* | Oral hygiene Poor | 1.01 | 0.013 | 0.164 |
| *Capnocytophaga granulosa* | Oral hygiene Poor | 1.10 | 0.015 | 0.177 |
| *Prevotella oulorum* | Oral hygiene Poor | 1.04 | 0.020 | 0.200 |


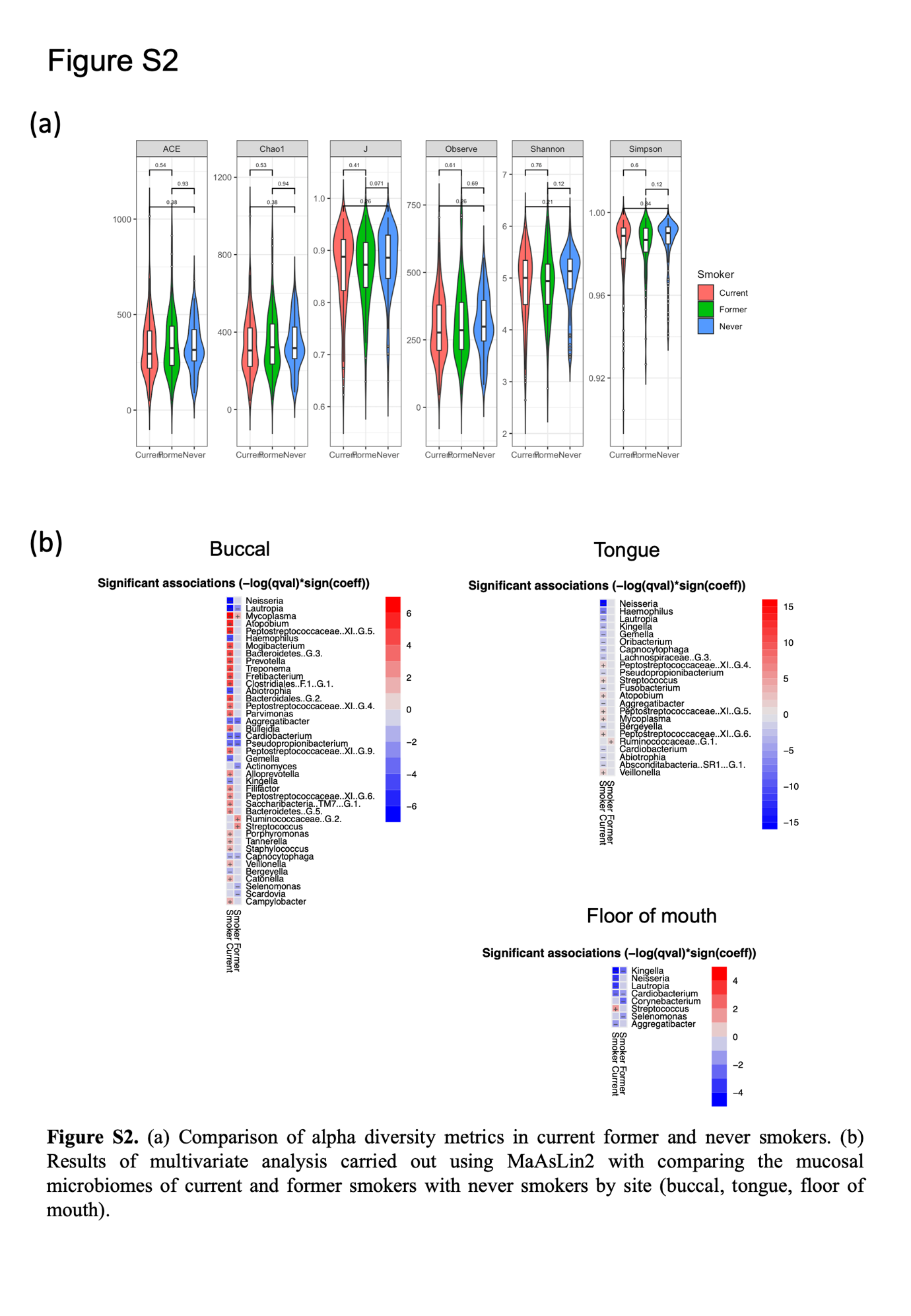


**Table S4.** Pairwise PERMANOVA results for smoking status.

| **Smoking status comparison** | **p value** | **Adjusted p value** |
| --- | --- | --- |
| Never vs current | 0.0001 | 0.0003 |
| Never vs former | 0.002 | 0.002 |
| Current vs former | 0.000 | 0.0003 |

**Table S5.** Species with significant changes in abundance associated with smoking on MaAsLin2 analysis (negative coefficient values denote reduced abundance)

| **Species** | **Variable** | **Coefficient** | **P value** | **Padj** |
| --- | --- | --- | --- | --- |
| *Neisseria perflava flavescens* | Current smoker | -1.23 | 4.81E-11 | 1.31E-08 |
| *Lautropia mirabilis* | Current smoker | -0.72 | 7.02E-11 | 1.31E-08 |
| *Rothia aeria* | Current smoker | -0.68 | 4.03E-09 | 5.03E-07 |
| *Haemophilus parainfluenzae* | Current smoker | -0.72 | 6.17E-09 | 5.77E-07 |
| *Streptococcus australis* | Current smoker | -0.67 | 6.45E-08 | 4.82E-06 |
| *Streptococcus* sp.*HMT066* sp. *HMT074* | Current smoker | -0.73 | 2.74E-07 | 0.00002 |
| *Streptococcus parasanguinis clade 411:721* | Current smoker | 0.74 | 5.75E-07 | 0.00003 |
| *Stomatobaculum* sp. *HMT097* | Current smoker | -0.41 | 8.53E-07 | 0.00004 |
| *Kingella oralis* | Current smoker | -0.64 | 1.45E-06 | 0.00005 |
| *Prevotella pleuritidis* | Current smoker | 0.51 | 1.32E-06 | 0.00005 |
| *Abiotrophia defectiva* | Current smoker | -0.32 | 4.08E-06 | 0.0001 |
| *Peptostreptococcaceae XI G 5 saphenum* | Current smoker | 0.49 | 4.59E-06 | 0.0001 |
| *Bergeyella* sp. *HMT322* | Current smoker | -0.38 | 5.01E-06 | 0.0001 |
| *Streptococcus oralis* subsp*. tigurinus clade 071* | Current smoker | 0.52 | 6.79E-06 | 0.0002 |
| *Streptococcus sanguinis* | Current smoker | -0.52 | 9.61E-06 | 0.0002 |
| *Cardiobacterium hominis* | Current smoker | -0.28 | 0.00001 | 0.0003 |
| *Clostridiales F 1 G 1 bacterium HMT093* | Current smoker | 0.23 | 0.00001 | 0.0003 |
| *Atopobium parvulum* | Current smoker | 0.60 | 0.00002 | 0.0004 |
| *Neisseria oralis* | Current smoker | -0.31 | 0.00002 | 0.0004 |
| *Actinomyces* sp. *HMT169* | Current smoker | -0.47 | 0.00002 | 0.0004 |
| *Capnocytophaga sputigena* | Current smoker | -0.41 | 0.00002 | 0.0004 |
| *Mycoplasma faucium* | Current smoker | 0.34 | 0.0001 | 0.002 |
| *Fusobacterium periodonticum hwasookii* | Current smoker | -0.48 | 0.0001 | 0.002 |
| *Mogibacterium timidum* | Current smoker | 0.34 | 0.0001 | 0.002 |
| *Stomatobaculum longum* | Current smoker | 0.32 | 0.0002 | 0.003 |
| *Neisseria macacae flava mucosa sicca* | Current smoker | -0.50 | 0.0003 | 0.004 |
| *Streptococcus* sp. *HMT066* | Current smoker | -0.50 | 0.0004 | 0.005 |
| *Streptococcus intermedius* | Current smoker | -0.31 | 0.0005 | 0.006 |
| *Streptococcus thermophilus salivarius* | Current smoker | 0.23 | 0.0005 | 0.006 |
| *Prevotella histicola* | Current smoker | 0.59 | 0.0006 | 0.007 |
| *Actinomyces lingnae* | Current smoker | 0.38 | 0.0006 | 0.007 |
| *Porphyromonas endodontalis* | Current smoker | 0.43 | 0.0007 | 0.007 |
| *Actinomyces graevenitzii* | Current smoker | 0.49 | 0.0007 | 0.008 |
| *Peptostreptococcaceae XI G 4 bacterium HMT369* | Current smoker | 0.27 | 0.0008 | 0.009 |
| *Prevotella* sp. *HMT313* | Current smoker | 0.41 | 0.002 | 0.02 |
| *Streptococcus* sp. *HMT057* | Current smoker | 0.41 | 0.002 | 0.02 |
| *Aggregatibacter* sp. *HMT512* | Current smoker | -0.20 | 0.003 | 0.02 |
| *Bacteroidales G 2 bacterium HMT274* | Current smoker | 0.27 | 0.003 | 0.02 |
| *Fusobacterium* sp. *HMT203* | Current smoker | 0.21 | 0.004 | 0.03 |
| *Selenomonas artemidis* | Current smoker | -0.24 | 0.004 | 0.03 |
| *Capnocytophaga gingivalis* | Current smoker | -0.21 | 0.004 | 0.03 |
| *Treponema* sp. *HMT237* | Current smoker | 0.20 | 0.004 | 0.03 |
| *Prevotella* sp. *HMT526* | Current smoker | 0.19 | 0.004 | 0.03 |
| *Parvimonas micra* | Current smoker | 0.38 | 0.004 | 0.03 |
| *Filifactor alocis* | Current smoker | 0.36 | 0.004 | 0.03 |
| *Porphyromonas catoniae* | Current smoker | -0.12 | 0.005 | 0.03 |
| *Streptococcus oralis* subsp*. tigurinus clade 070* | Current smoker | 0.33 | 0.005 | 0.03 |
| *Lachnoanaerobaculum umeaense* | Current smoker | -0.19 | 0.005 | 0.03 |
| *Gemella morbillorum* | Current smoker | -0.31 | 0.005 | 0.03 |
| *Veillonella denticariosi* | Current smoker | 0.33 | 0.005 | 0.03 |
| *Peptostreptococcaceae XI G 6 nodatum* | Current smoker | 0.22 | 0.006 | 0.04 |
| *Aggregatibacter* sp. *HMT458* | Current smoker | -0.19 | 0.006 | 0.04 |
| *Streptococcus* sp.*HMT064* sp. *HMT423 mitis* | Current smoker | 0.23 | 0.006 | 0.04 |
| *Fusobacterium nucleatum* subsp. *vincentii* | Current smoker | 0.34 | 0.007 | 0.04 |
| *Campylobacter concisus* | Current smoker | -0.30 | 0.007 | 0.04 |
| *Neisseria elongata* | Current smoker | -0.21 | 0.008 | 0.05 |
| *Alloprevotella tannerae* | Current smoker | 0.37 | 0.009 | 0.05 |
| *Streptococcus intermedius* | Former smoker | -0.40 | 0.00006 | 0.0010 |
| *Actinomyces* sp. *HMT169* | Former smoker | -0.45 | 0.0003 | 0.004 |
| *Streptococcus gordonii* | Former smoker | -0.60 | 0.0009 | 0.009 |
| *Selenomonas artemidis* | Former smoker | -0.31 | 0.001 | 0.01 |
| *Cardiobacterium hominis* | Former smoker | -0.22 | 0.002 | 0.02 |
| *Streptococcus* *oralis infantis* | Former smoker | 0.28 | 0.003 | 0.02 |
| *Actinomyces* sp. *HMT175* | Former smoker | -0.32 | 0.006 | 0.04 |


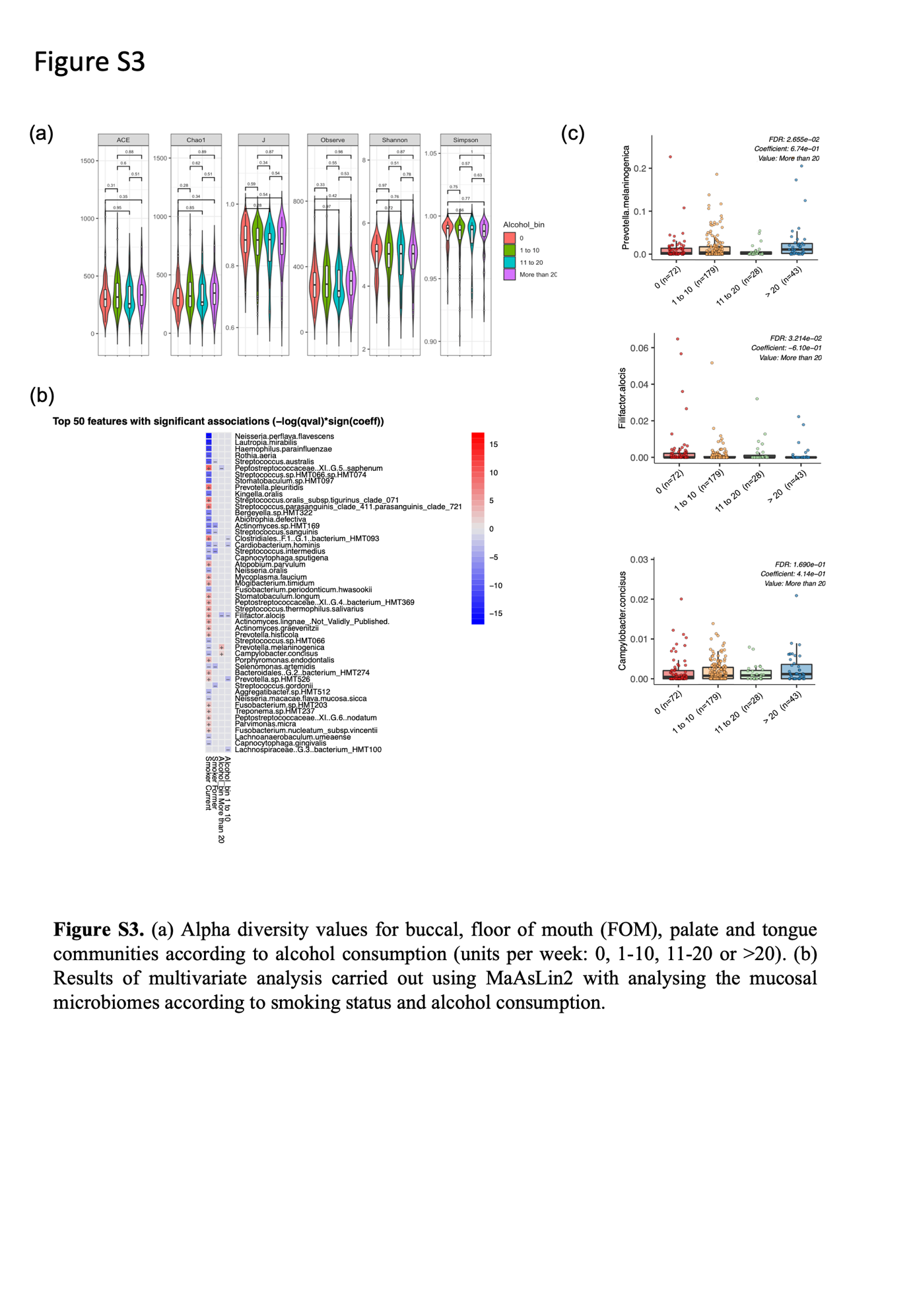


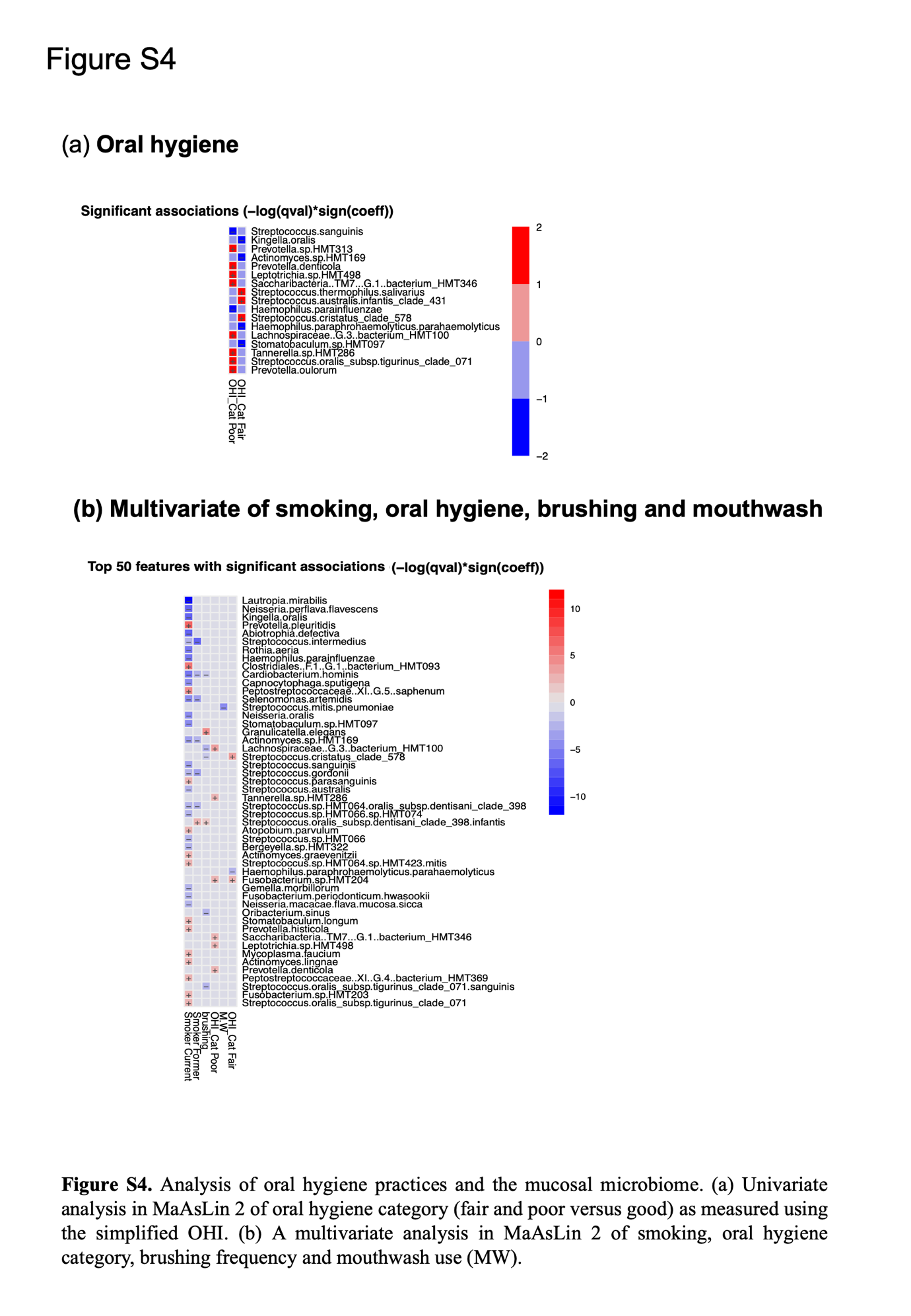


**Table S6.**  Species with significant changes in abundance associated oral hygiene on MaAsLin2 analysis (negative values denote reduced abundance)

| **Species** | **Variable** | **Coefficient** | **P value** | **Padj** |
| --- | --- | --- | --- | --- |
| *Granulicatella.elegans* | Brushing ≤1 Day | 1.59 | 0.00 | 0.02 |
| *Oribacterium.sinus* | Brushing ≤1 Day | -1.56 | 0.00 | 0.08 |
| *Streptococcus.oralis_subsp.tigurinus_clade_071.sanguinis* | Brushing ≤1 Day | -1.21 | 0.01 | 0.11 |
| *Lachnospiraceae..G.3..bacterium_HMT100* | Brushing ≤1 Day | -1.12 | 0.01 | 0.12 |
| *Prevotella.sp.HMT526* | Brushing ≤1 Day | 0.77 | 0.01 | 0.15 |
| *Streptococcus.oralis.infantis* | Brushing ≤1 Day | 0.91 | 0.01 | 0.16 |
| *Haemophilus.parahaemolyticus* | Brushing ≤1 Day | 1.22 | 0.01 | 0.16 |
| *Streptococcus.oralis:mitis* | Brushing ≤1 Day | 0.67 | 0.01 | 0.17 |
| *Corynebacterium.durum* | Brushing ≤1 Day | -0.78 | 0.01 | 0.17 |
| *Streptococcus.cristatus_clade_578* | Brushing ≤1 Day | -1.30 | 0.01 | 0.18 |
| *Cardiobacterium.hominis* | Brushing ≤1 Day | -0.74 | 0.02 | 0.22 |
| *Lachnoanaerobaculum.orale* | Brushing ≤1 Day | -0.51 | 0.02 | 0.23 |
| *Lachnoanaerobaculum.umeaense.saburreum* | Brushing ≤1 Day | -0.70 | 0.02 | 0.23 |
| *Streptococcus.mitis.pneumoniae* | Mouthwash Yes | -2.26 | 0.00 | 0.01 |
| *Prevotella.pallens* | Mouthwash Yes | -0.86 | 0.01 | 0.14 |
| *Veillonella.sp.HMT780* | Mouthwash Yes | -0.54 | 0.02 | 0.22 |
| *Lachnoanaerobaculum.umeaense.saburreum* | Mouthwash Yes | -0.50 | 0.02 | 0.25 |
| *Streptococcus.cristatus_clade_578* | Oral Hygiene Fair | 1.58 | 0.00 | 0.02 |
| *Haemophilus.paraphrohaemolyticus.parahaemolyticus* | Oral Hygiene Fair | -0.77 | 0.00 | 0.07 |
| *Fusobacterium.sp.HMT204* | Oral Hygiene Fair | 1.06 | 0.00 | 0.07 |
| *Streptococcus.australis.infantis_clade_431* | Oral Hygiene Fair | 0.92 | 0.01 | 0.20 |
| *Corynebacterium.durum* | Oral Hygiene Fair | 0.63 | 0.02 | 0.25 |
| *Streptococcus.thermophilus.salivarius* | Oral Hygiene Fair | 0.46 | 0.02 | 0.25 |
| *Lachnospiraceae..G.3..bacterium_HMT100* | Oral Hygiene Poor | 1.52 | 0.00 | 0.02 |
| *Tannerella.sp.HMT286* | Oral Hygiene Poor | 1.43 | 0.00 | 0.06 |
| *Fusobacterium.sp.HMT204* | Oral Hygiene Poor | 1.26 | 0.00 | 0.08 |
| *Saccharibacteria..TM7...G.1..bacterium_HMT346* | Oral Hygiene Poor | 1.02 | 0.00 | 0.09 |
| *Leptotrichia.sp.HMT498* | Oral Hygiene Poor | 1.20 | 0.00 | 0.09 |
| *Prevotella.denticola* | Oral Hygiene Poor | 1.58 | 0.00 | 0.09 |
| *Capnocytophaga.granulosa* | Oral Hygiene Poor | 1.03 | 0.02 | 0.22 |
| *Prevotella.sp.HMT313* | Oral Hygiene Poor | 1.40 | 0.02 | 0.25 |
| *Prevotella.oulorum* | Oral Hygiene Poor | 0.93 | 0.02 | 0.25 |


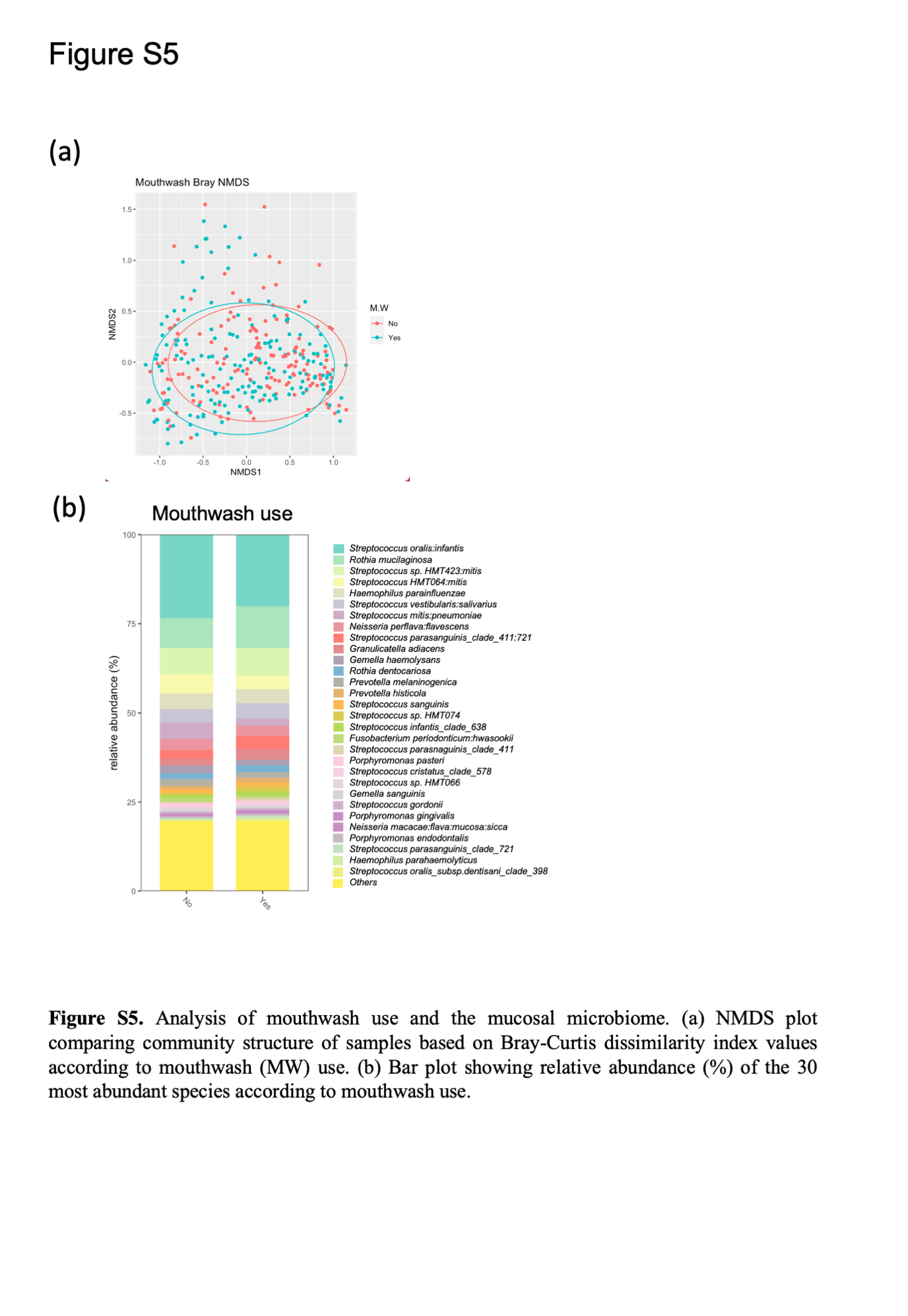


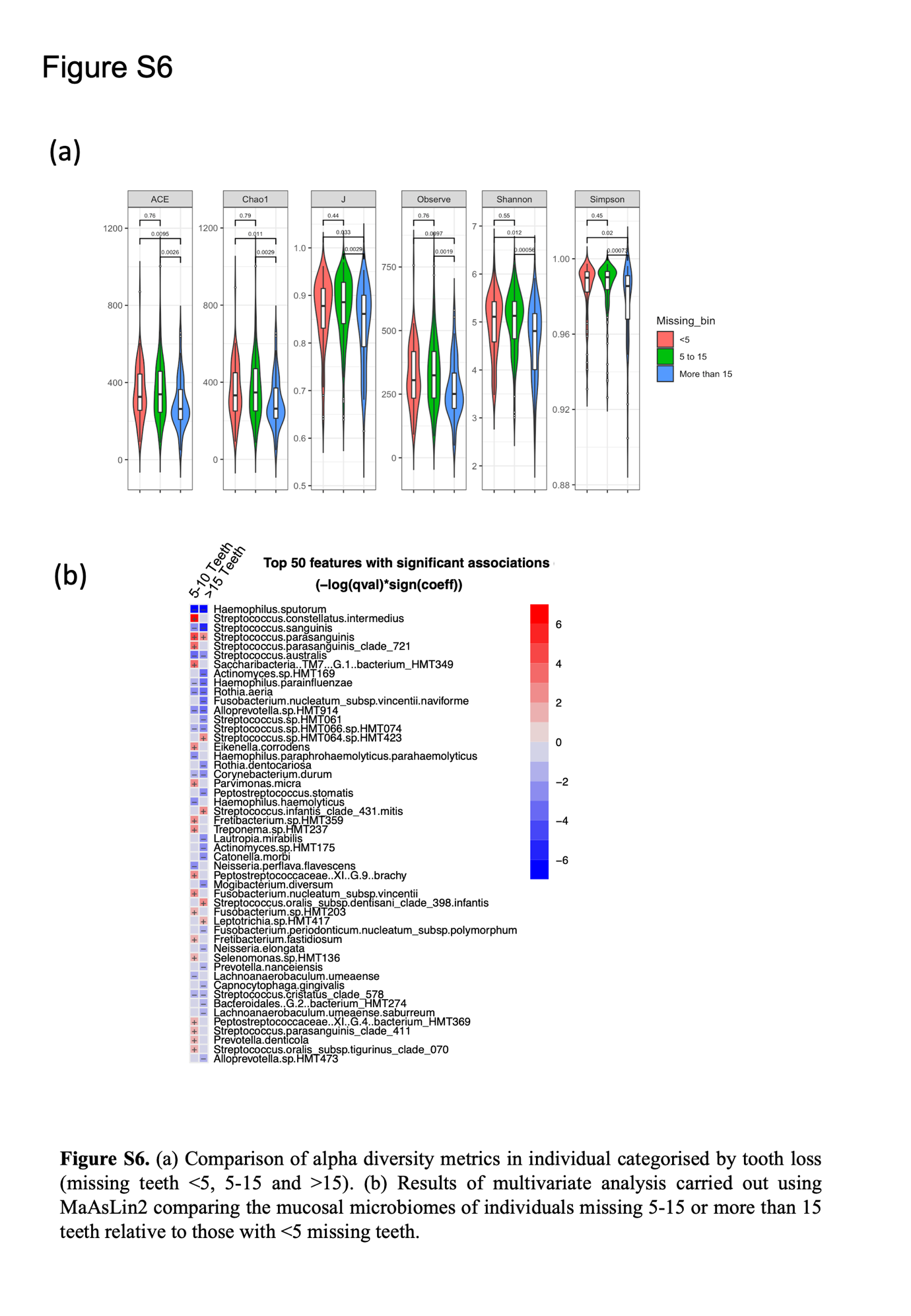


**Table S7.** Results of MaAsLin2 analysis showing species with significant changes in abundance associated with number of missing teeth

| **Species** | **Tooth loss** | **Coefficient** | **P value** | **Padj** |
| --- | --- | --- | --- | --- |
| *Haemophilus.sputorum* | 5 to 15 | -1.336 | 0.000 | 0.001 |
| *Streptococcus.constellatus.intermedius* | 5 to 15 | 2.116 | 0.000 | 0.001 |
| *Streptococcus.parasanguinis* | 5 to 15 | 1.931 | 0.000 | 0.017 |
| *Streptococcus.parasanguinis_clade_721* | 5 to 15 | 1.666 | 0.001 | 0.034 |
| *Streptococcus.australis* | 5 to 15 | -1.467 | 0.001 | 0.040 |
| *Saccharibacteria..TM7...G.1..bacterium_HMT349* | 5 to 15 | 0.861 | 0.001 | 0.040 |
| *Eikenella.corrodens* | 5 to 15 | 0.874 | 0.003 | 0.072 |
| *Haemophilus.paraphrohaemolyticus.parahaemolyticus* | 5 to 15 | -0.700 | 0.003 | 0.072 |
| *Rothia.aeria* | 5 to 15 | -1.211 | 0.004 | 0.078 |
| *Parvimonas.micra* | 5 to 15 | 1.259 | 0.006 | 0.085 |
| *Haemophilus.haemolyticus* | 5 to 15 | -0.927 | 0.006 | 0.085 |
| *Fretibacterium.sp.HMT359* | 5 to 15 | 0.694 | 0.005 | 0.085 |
| *Treponema.sp.HMT237* | 5 to 15 | 0.658 | 0.006 | 0.085 |
| *Alloprevotella.sp.HMT914* | 5 to 15 | -0.602 | 0.007 | 0.088 |
| *Haemophilus.sputorum* | >15 | -1.789 | 0.000 | 0.001 |
| *Streptococcus.sanguinis* | >15 | -2.145 | 0.000 | 0.006 |
| *Actinomyces.sp.HMT169* | >15 | -1.776 | 0.001 | 0.044 |
| *Haemophilus.parainfluenzae* | >15 | -1.735 | 0.002 | 0.047 |
| *Rothia.aeria* | >15 | -1.736 | 0.001 | 0.047 |
| *Fusobacterium.nucleatum_subsp.vincentii.naviforme* | >15 | -1.918 | 0.001 | 0.047 |
| *Alloprevotella.sp.HMT914* | >15 | -0.919 | 0.002 | 0.047 |
| *Streptococcus.sp.HMT061* | >15 | -1.613 | 0.002 | 0.061 |
| *Streptococcus.sp.HMT066.sp.HMT074* | >15 | -1.952 | 0.003 | 0.065 |
| *Streptococcus.sp.HMT064.sp.HMT423* | >15 | 0.814 | 0.003 | 0.072 |
| *Rothia.dentocariosa* | >15 | -1.603 | 0.005 | 0.084 |
| *Corynebacterium.durum* | >15 | -0.890 | 0.005 | 0.084 |
| *Streptococcus.australis* | >15 | -1.552 | 0.006 | 0.085 |
| *Peptostreptococcus.stomatis* | >15 | -1.270 | 0.005 | 0.085 |
| *Streptococcus.infantis_clade_431.mitis* | >15 | 1.071 | 0.006 | 0.085 |
| *Lautropia.mirabilis* | >15 | -1.458 | 0.007 | 0.088 |
| *Actinomyces.sp.HMT175* | >15 | -1.343 | 0.007 | 0.088 |


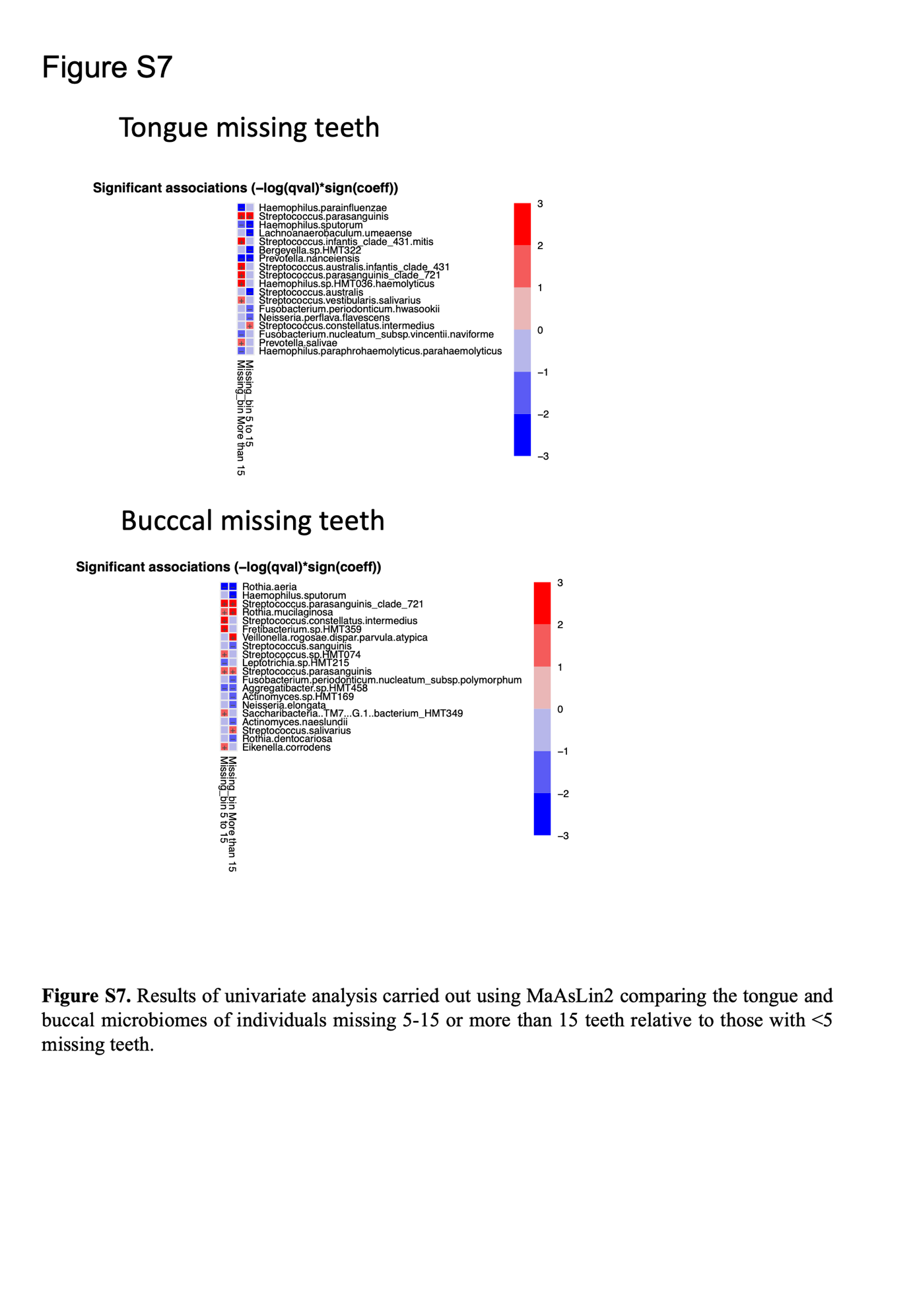


**
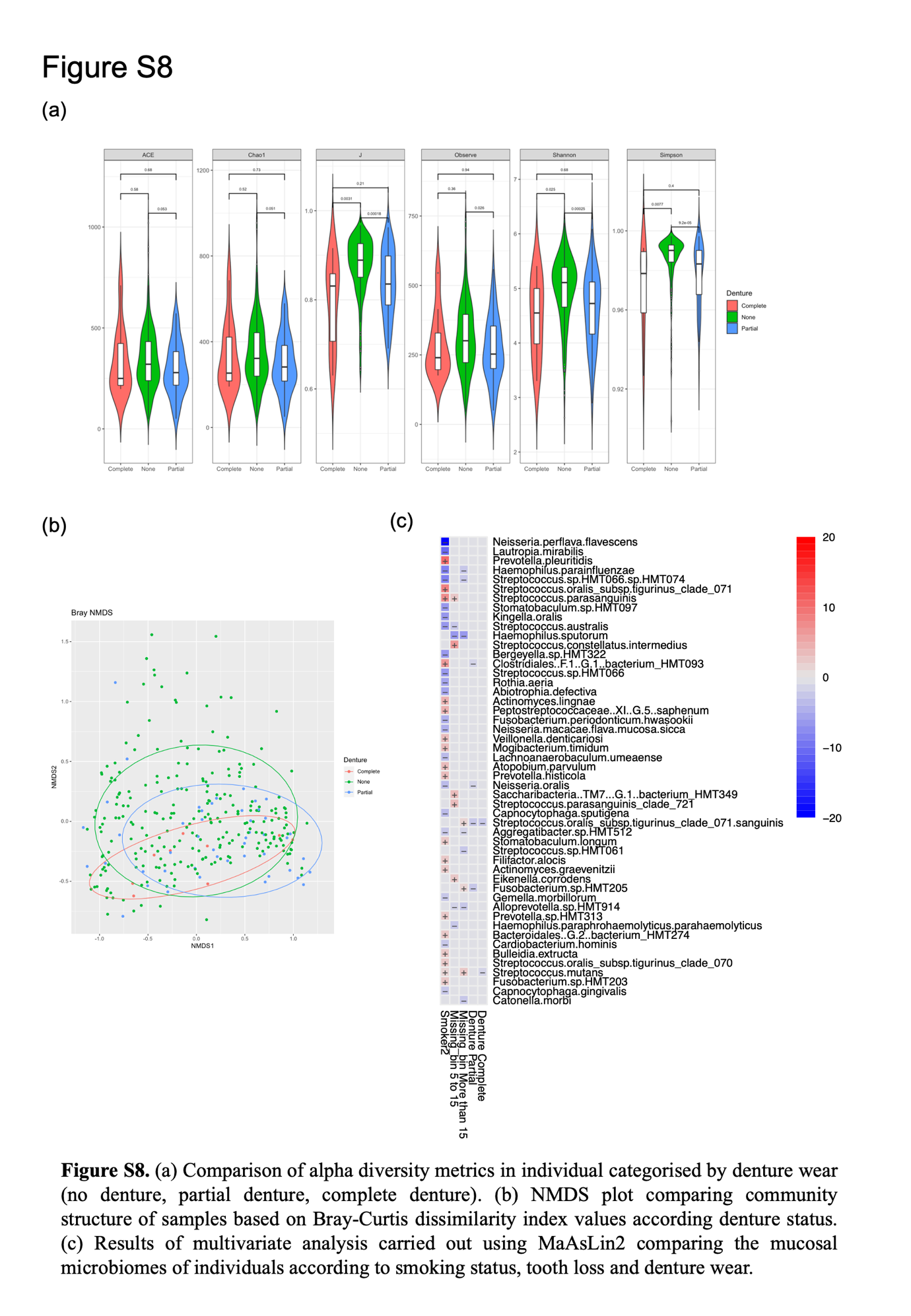
**

**
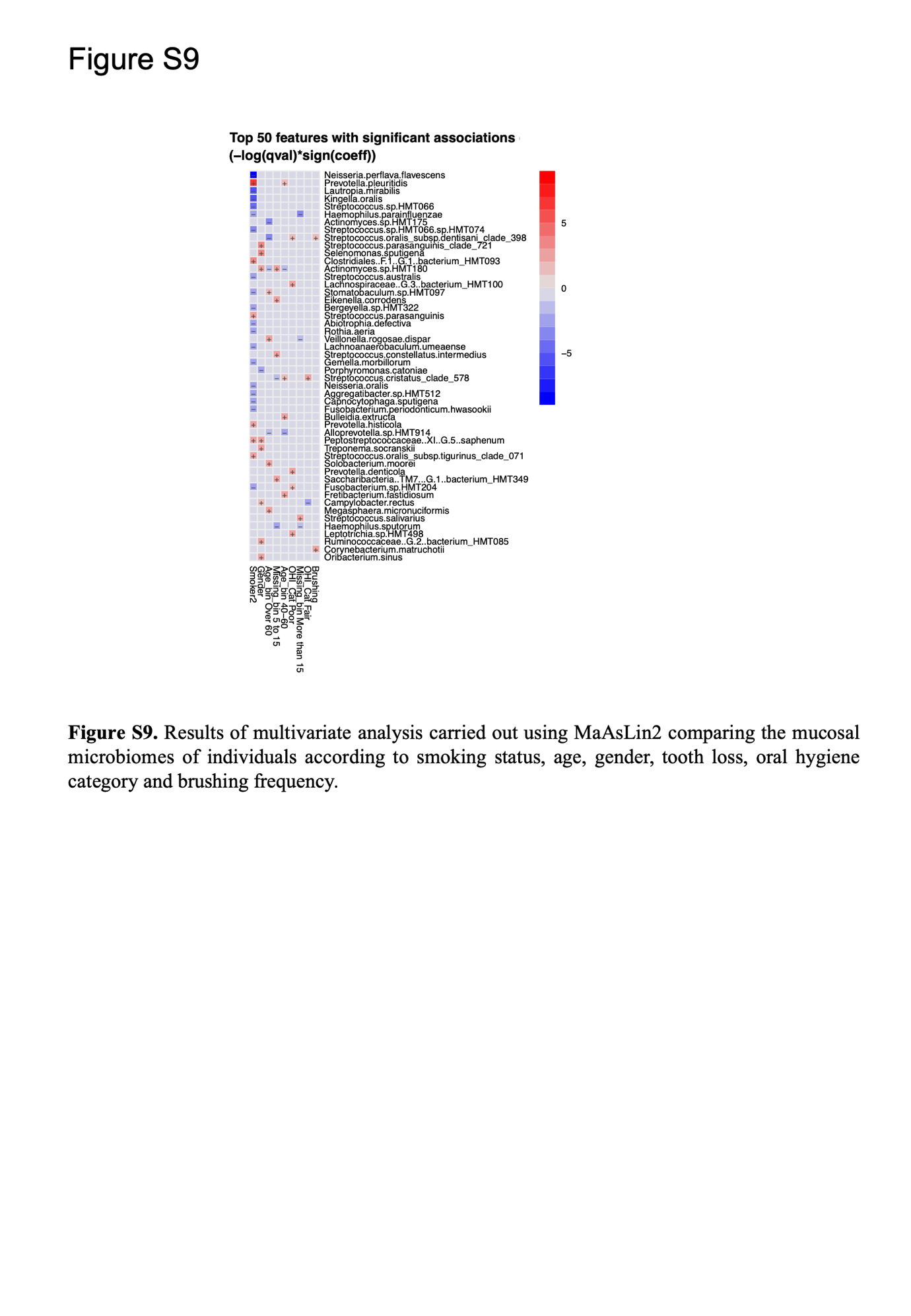
**

**Table S8.** Species with significant changes in abundance associated with both age and gender (negative coefficient value denotes reduced abundance)

| **Species** | **Variable** | **Coefficient** | **P value** | **Padj** |
| --- | --- | --- | --- | --- |
| *Actinomyces.sp.HMT175* | Age >60 | -2.467985 | 0.00015477 | 0.04134497 |
| *Streptococcus.oralis_subsp.dentisani_clade_398* | Age >60 | -2.6757532 | 0.00025899 | 0.04402809 |
| *Veillonella.rogosae.dispar* | Age >60 | 2.8331382 | 0.00093918 | 0.0763598 |
| *Bulleidia.extructa* | Age 40-60 | 0.83176553 | 0.00183988 | 0.10119343 |
| *Alloprevotella.sp.HMT914* | Age 40-60 | -0.9789704 | 0.00194322 | 0.10354372 |
| *Streptococcus.parasanguinis_clade_721* | Male Gender | 1.69864449 | 0.00023787 | 0.04402809 |
| *Selenomonas.sputigena* | Male Gender | 1.20109971 | 0.00024984 | 0.04402809 |
| *Actinomyces.sp.HMT180* | Male Gender | 0.7174822 | 0.00040227 | 0.05786463 |
| *Porphyromonas.catoniae* | Male Gender | -0.5147154 | 0.00141386 | 0.09442543 |
| *Peptostreptococcaceae..XI..G.5..saphenum* | Male Gender | 1.12154478 | 0.00219686 | 0.11074696 |
| *Treponema.socranskii* | Male Gender | 0.76301509 | 0.00225047 | 0.11074696 |
